# Supplementary material for: Recommendations of the Netzwerk Kindersimulation for the Implementation of Simulation-Based Pediatric Team Trainings: A Delphi Process
Source: Children (Basel). 2023 Jun 16;10(6):1068. doi: 10.3390/children10061068 (PMC10297481; doi:10.3390/children10061068)
Supplement: Supplementary file 1 [file children-10-01068-s001.zip › children-2415882-supplementary/Quality criteria/Supplementary Materials File S1. Qualitätskriterien_dt_Erste Auflage.pdf]

Empfehlungen

des Netzwerks Kindersimulation e.V. für die Durchführung simulationsbasierter pädiatrischer Teamtrainings

Ruth M. Löllgen, Lukas P. Mileder, Katharina Bibl, Christian Dörfler, Annika Paulun, Jasmin Rupp, Benedikt Sandmeyer, Alex Staffler, Michael Wagner, Ellen Heimberg

Erste Auflage Juni 2020

NETZWERK KINDERSIMULATION

Kontakt

vorstand@netzwerk-kindersimulation.org

Universitätsklinikum Tübingen
Kinderklinik
Hoppe-Seyler-Str. 1
72076 Tübingen
Deutschland

www.netzwerk-kindersimulation.org

NETZWERK
KINDERSIMULATION

Abkürzungsverzeichnis

AHA	American Heart Association
ALS	Advanced Life Support
BLS	Basic Life Support
CRM	Crisis Resource Management/ Crew Resource Management
DASH	Debriefing Assessment for Simulation in Healthcare
EPALS	European Pediatric Advanced Life Support
ERC	European Resuscitation Council
HRO	High Reliability Organisation
NLS	Newborn Life Support
NRP	Neonatal Resuscitation Program
NTS	Non-Technical Skills (Nicht-technische Fertigkeiten)
OSAD	Objective Structured Assessment of Debriefing
PALS	Pediatric Advanced Life Support
SOP	Standard Operating Procedure
OSCE	Objective Structured Clinical Examination
CIRS	Critical Incident Reporting System

1) **PRÄAMBEL** 3

2) **ALLGEMEINE LERNZIELE EINES SIMULATIONSBASIERTEN TEAMTRAININGS** 4

3) **QUALIFIKATION DER SIMULATIONSTRAINER** 4

a) MEDIZINISCH-FACHLICHE VORAUSSETZUNG 4
b) NOTFALLAUSBILDUNG 4
c) SIMULATIONSTRAINER-AUSBILDUNG 5
d) KENNTNISSE IN NICHT-MEDIZINISCHEN FERTIGKEITEN („NON-TECHNICAL SKILLS“) 5
e) SIMULATIONS-MENTORING 5
f) KONTINUIERLICHE WEITERBILDUNG IN SIMULATION 6
g) MINDESTANFORDERUNG ANZAHL SIMULATIONSTRAININGS 6

4) **RAHMENBEDINGUNGEN FÜR EINE EFFEKTIVE LERNUMGEBUNG** 6

a) VERTRAULICHKEIT 6
b) UMGANG MIT VIDEOS 7
c) UMGANG MIT FOTOS 7
d) ÖFFENTLICHKEITSARBEIT/ PRESSE 7
e) LERNKLIMA 7
f) „TOD“ DES SIMULATORS 8

5) **SIMULATIONSUMGEBUNG** 8

a) RÄUMLICHKEITEN 8
b) MEDIZINISCHES EQUIPMENT IN DER SIMULATION 8
c) SIMULATOREN 9
d) STANDARDISIERTE PATIENTEN 9
e) AUDIO-VIDEO ANLAGE 9

6) **PERSONELLE RESSOURCEN** 9

a) TEILNEHMER-TEAM 9
b) SIMULATIONSTRAINER 10

7) **SZENARIEN** 11

a) LERNZIELE 11
b) SZENARIENENTWICKLUNG 12
c) SZENARIENSKRIPT 12

8) **ABLAUF EINES TRAININGS** 13

a) VORBESPRECHUNG TRAINERTEAM 13
b) EINFÜHRUNG 13
c) FAMILIARISIERUNG MIT DEM SIMULATOR UND DER SIMULATIONSUMGEBUNG 13
d) BRIEFING DER TEILNEHMER (VORBESPRECHUNG) 13
e) DURCHFÜHRUNG DES SZENARIOS 14
f) DEBRIEFING (= NACHBESPRECHUNG) 14
g) DURCHFÜHRUNG WEITERER SZENARIEN UND DEBRIEFINGS 15
h) KURSEVALUATION 15
i) NACHBESPRECHUNG DES TRAINERTEAMS 15

9) **FEEDBACK UND EVALUATION** 15

a) FEEDBACK DER TEILNEHMER 15
b) NACHBESPRECHUNG DES TRAINERTEAMS 15
c) ZUSAMMENFASSUNG DES TRAININGS 16

10) **ANHANG** 17

11) **LITERATUR** 20

1) PRÄAMBEL

Das Netzwerk Kindersimulation e.V. hat folgende Empfehlungen erarbeitet, um einen einheitlichen Standard für die Durchführung simulationsbasierter Teamtrainings von pädiatrischen Notfällen und eine hohe Qualität der simulationsbasierten Aus-, Fort- und Weiterbildung in den deutschsprachigen Ländern gewährleisten zu können.

Die Empfehlungen orientieren sich, wo vorhanden, an der aktuellen Fachliteratur sowie an bestehenden Empfehlungen/Qualitätskriterien entsprechender Fachgesellschaften[1–3]. Aufgrund der in vielen Bereichen der simulationsbasierten Aus-, Fort- und Weiterbildung geringen wissenschaftlichen Evidenz wurden zahlreiche multiprofessionelle Experten in die Erstellung dieser multiprofessionellen Empfehlungen miteinbezogen, um das vorhandene Wissen und bestehende Erfahrungen in der Konzeption und Durchführung pädiatrischer Simulationstrainings bestmöglich miteinzubeziehen. Der Hauptfokus dieses Dokumentes sind simulationsbasierte Teamtrainings, womit sich diese Empfehlungen bewusst von jenen für andere Trainingsformen (siehe Tabelle 1) abgrenzen. Die Tabelle im Anhang enthält jedoch zur besseren Übersicht bzw. Vergleichbarkeit für alle Bereiche der Simulation zusammengefasste Empfehlungen für Mindeststandards.

Die vorliegenden Empfehlungen richten sich an alle Personen, die sich im Gesundheitswesen im Bereich der Präklinik, in der ambulanten Versorgung sowie innerklinisch (Kreißsaal, Kinder- und Neugeborenen-Intensivstation, Notfallstationen und Notaufnahmen, Operationssaal, Aufwachraum, Tageskliniken, Kinderstationen, usw.) in der pädiatrischen Aus-, Fort- und Weiterbildung mittels simulationsbasierten Teamtrainings engagieren oder dies anstreben. Zudem richten sich diese Empfehlungen auch an all jene Personen, die als Verantwortliche für den Bereich Simulation und/oder als Trainer aktiv sind. Generell sind die hier zusammengefassten Empfehlungen unabhängig vom Ort des Teamtrainings und beziehen sich sowohl auf Trainings in einem Simulationszentrum als auch auf Trainings „in situ", d.h. am realen Arbeitsplatz. Die Empfehlungen unterscheiden nicht zwischen den verschiedenen Realitätsgraden der eingesetzten Simulatoren und Simulationsobjekte.

Diese Empfehlungen beschreiben die für Simulationstrainings benötigte materielle und personelle Ausstattung sowie Inhalte und Aufbau von simulationsbasierten Teamtrainings. Ziel ist es, über eine Standardisierung dieser Trainings eine möglichst hohe Ausbildungsqualität und somit eine verbesserte Versorgung von kranken und verletzten Neugeborenen und Kindern sowie eine Erhöhung der Patientensicherheit zu erreichen.

Die folgenden Empfehlungen sollen als Grundlage für die Organisation, Durchführung und Qualitätssicherung dieser Trainings dienen. Konkrete Empfehlungen zu technischer Ausrüstung und ausführliche Inhalte, z.B. Szenarien sowie Techniken des Debriefings, sind nicht Teil der Empfehlungen. Generell sollte zur Aufrechterhaltung der Qualität von Simulationsprogrammen zumindest ein Verantwortlicher benannt werden, der die Einhaltung dieser Empfehlungen regelmäßig überprüft und ggf. korrigierende Maßnahmen einleitet.

Im Text wird eine Graduierung der Empfehlungen nach Expertenmeinung und wenn gegeben nach Evidenz angewandt. Dabei wird in absteigender Folge der Empfehlungsstärke in "soll", „sollte" und „kann" eingeteilt.

Im Text wurde bewusst im Sinne der einfacheren Lesbarkeit durchweg die männliche Geschlechtsform verwendet. Sofern nicht explizit angegeben, beziehen sich die personenbezogenen Angaben stets in gleichem Umfang auf alle Geschlechter.

Die Definition von allfälligen Fachbegriffen im Text wurde auf den Inhalt des Simulationswörterbuches im Gesundheitswesen (engl. „Healthcare Simulation Dictionary") der Society for Simulation in Healthcare™ (https://www.ssih.org/dictionary) gestützt[4].

Die folgenden Empfehlungen beziehen sich auf traditionelle simulationsbasierte Team Trainings und decken explizit nicht den Bereich der Tele-Simulation, e-Simulation, Simulation aus der Entfernung oder virtuelle Simulation ab, auch wenn viele Empfehlungen überlappend gelten können.

2) ALLGEMEINE LERNZIELE EINES SIMULATIONSBASIERTEN TEAMTRAININGS

Primäres Ziel von Simulationstrainings ist die Verbesserung der Patientensicherheit.

Dies wird durch hochqualitatives, an das Erfahrungsniveau der Teilnehmer angepasstes Training erreicht, welches die Verbesserung medizinisch-technischer, kognitiver und sozialer Fertigkeiten sowie die Einstellung im Umgang mit komplexen medizinischen Situationen beinhaltet.

Neben kommunikativen Aspekten sollte das Erlernen folgender Grundsätze sichergestellt werden[5]:

- Situationsbewusstsein
- Aufgabenmanagement
- Teamarbeit
- Entscheidungsfindung

3) QUALIFIKATION DER SIMULATIONSTRAINER

Der Auswahl von Simulationstrainern kommt eine große Bedeutung zu, da deren Aufgabe einen hohen Anspruch an fachliche und soziale Kompetenz stellt. Die folgenden Qualifikationen sollen für mindestens einen der anwesenden Trainer in einem Training gelten. Der zweite Trainer kann sich in allen Qualitäten noch in Weiterbildung befinden.

a) MEDIZINISCH-FACHLICHE VORAUSSETZUNG

Es soll eine mindestens dreijährige praktische Erfahrung (in Vollzeit oder Teilzeitäquivalent) in der Kinder- und Jugendmedizin (Pädiatrie, Kinderchirurgie) oder eine mindestens dreijährige Erfahrung in der medizinischen Versorgung von Neugeborenen und Kindern (Kinderkrankenpflege, Hebammen, Anästhesie, Rettungsdienst) vorliegen. Vorteilhaft ist eine spezielle Expertise im Bereich Neonatologie, pädiatrischer Intensivmedizin und/ oder Kindernotfallmedizin.

b) NOTFALLAUSBILDUNG

Eine erfolgreiche Teilnahme an einem zertifizierten Kindernotfallkurs (z.B. European Pediatric Advanced Life Support (EPALS) des European Resuscitation Council (ERC) oder Pediatric Advanced Life Support (PALS) der American Heart Association (AHA) für pädiatrische Notfalltrainings bzw. Newborn Life Support (NLS) des ERC oder Neonatal Resuscitation Program (NRP) der AHA für neonatologische Notfalltrainings) oder vergleichbare klinikinterne, zertifizierte Kursformate zu erweiterten Notfallmaßnahmen beim Kind sowie die

Aufrechterhaltung des Providerstatus durch die regelmäßige Teilnahme an entsprechenden Auffrischungskursen ist Grundvoraussetzung.

Es muss sichergestellt werden, dass Szenarieninhalte und Debriefings den aktuell publizierten Leitlinien entsprechen und bei Aktualisierungen der Leitlinien diese zeitnah angepasst werden.

c) SIMULATIONSTRAINER-AUSBILDUNG

Die Teilnahme an einem Train-the-Trainer-Kurs für Simulation mit Inhalten des Crisis Resource Managements (CRM[6,7] = Prinzipien des individuellen und vor allem gemeinsamen Handelns in Routine- und Krisensituationen, die effektives Entscheiden und Zusammenarbeiten ermöglichen), Werkzeugen zur Verbesserung der Patientensicherheit, des Szenariendesigns, theoretischen Inhalten zu Debriefings und der praktischen Durchführung von Debriefings soll erfolgt sein. Dabei sollen sowohl Szenarienentwicklung, -vorbereitung und -durchführung und Debriefing jeweils mindestens einmal aktiv durchgeführt und dazu Feedback von den Kurstrainern erhalten worden sein.

d) KENNTNISSE IN NICHT-MEDIZINISCHEN FERTIGKEITEN („NON-TECHNICAL SKILLS")

Der Simulationstrainer soll mit den nicht-medizinischen Fertigkeiten („Non-Technical Skills", NTS) vertraut sein. Dazu gehören zum Beispiel: 15 Prinzipien des CRM[6,7], „Speaking-Up"[8], Aufgabenverteilung in Teams (Teamleiter und Teammitglieder), Durchführung von Team-Reflexionen[9,10] (STOP-Sequenzen, „10 Sekunden für 10 Minuten"[6]), effektive Kommunikationsstrategien[11,12] (z.B. „Closed-Loop"-Kommunikation), korrekte Anwendung von Checklisten, Erarbeiten gemeinsamer mentaler Modelle.[4,5]

Des Weiteren sollten grundlegende Aspekte zu folgenden Themen berücksichtigt bzw. sich angeeignet werden:

Werkzeuge zur Verbesserung der Patientensicherheit, Standardisierung („Standard Operating Procedures", SOP), Berichterstattung über kritische Ereignisse (engl. „Critical Incident Reporting System") sowie systemische Sicherheit in Organisationen mit hoher Zuverlässigkeit (engl. „High Reliability Organisation", HRO).

e) SIMULATIONS-MENTORING

Nach der Absolvierung eines Train-the-Trainer Kurses sollte die Vertiefung der folgenden Bereiche durch einen erfahrenen Mentor begleitet werden. Wenn im eigenen Umfeld diese Möglichkeit nicht vorhanden ist, kann über Experten des Netzwerks Kindersimulation eine Hospitation realisiert werden. Die publizierte Evidenz bezüglich Dauer, Häufigkeit und Ablauf eines solchen Mentorings ist noch knapp, aus unserer eigenen, nicht publizierten Erfahrung empfehlen wir jedoch regelmäßige gemeinsame Simulationstrainings mit einem erfahrenen Simulationstrainer und die (Nach-) Besprechung folgender Inhalte:

- Auswahl und Vorbereitung von Szenarien
- Einführung in das Simulationstraining
- Briefing von Statisten (engl. „Extra") oder Verbündeten (engl. „Confederate"[4]): Schauspieler, standardisierte Patienten = Personen außer dem Patienten/der Patientin, die skriptbasiert an einer Simulation mitwirken, um zusätzlichen Realismus oder zusätzliche Informationen für die Lernenden zu vermitteln)
- Familiarisierung

- Simulatorsteuerung und Technik
- Leitung eines Simulationstrainings
- Co-Debriefing
- Debriefing

f) KONTINUIERLICHE WEITERBILDUNG IN SIMULATION

Zur Aufrechterhaltung und Weiterentwicklung der Kenntnisse soll eine zumindest jährliche Fortbildung zum Thema Simulation und/oder Debriefing (internes oder externes Trainer-Peer-Feedback, Telesimulation, Hospitation in anderen Häusern, Workshops, Train-the-Trainer-Kurse, simulationsspezifische Kongresse/Fortbildungen, usw.) von mindestens vier Stunden absolviert werden. Eine Mitgliedschaft im Netzwerk Kindersimulation ist wünschenswert.

g) MINDESTANFORDERUNG ANZAHL SIMULATIONSTRAININGS

Zur Aufrechterhaltung der Lehrqualität sollen regelmäßig Szenarien inklusive Debriefings durchgeführt werden. Eine Anzahl von mindestens 10 Szenarien + Debriefings pro Jahr sollte angestrebt werden.

4) RAHMENBEDINGUNGEN FÜR EINE EFFEKTIVE LERNUMGEBUNG

Um den Teilnehmern ein effektives Lernen zu ermöglichen, ist eine sichere Lernumgebung erforderlich.[13–15] Deshalb muss für die Durchführung von simulationsbasierten Teamtrainings und Debriefings ein „geschützter Raum" geschaffen werden, der den Teilnehmern das Gefühl von Sicherheit erlaubt. Um diese „psychologische Sicherheit" für die Teilnehmer zu gewährleisten, müssen folgende Grundregeln beachtet werden.[16]

a) VERTRAULICHKEIT

i. Bei der Einführung des Kurses, in jedem Fall aber vor Beginn der Szenarien müssen die wichtigsten Regeln mit den Teilnehmern besprochen werden. Eventuell können diese auch zusätzlich vor Beginn des Kurses schriftlich mitgeteilt werden.

ii. Den Teilnehmern muss zugesichert werden, dass Inhalte über Leistungen von Teilnehmern niemandem außerhalb des Simulationstrainings kommuniziert werden (insbesondere nicht Vorgesetzten, Verwaltung und/oder Klinikleitung). Die einzigen Inhalte, die kommuniziert werden sollen, sind Probleme systemischen Ursprungs, z.B. technische, logistische oder Probleme mit der Ausrüstung. Diese Inhalte müssen losgelöst von einzelnen Personen weitergegeben werden. Eine Ausnahme, aber nicht Umfang dieser Empfehlungen bilden z.B. simulationsbasierte Beurteilungen von Studierenden oder Absolventen einer Facharzt-, Fachpflege-, Rettungssanitäter-, Hebammenprüfung etc., bei denen Leistungen der Teilnehmer transparent an die Prüfungskommission vermittelt werden, z.B. im Rahmen eines OSCE („Objective Structured Clinical Examination"[4]).

iii. Inhalte der Szenarien und der Debriefings müssen vertraulich behandelt werden. Dies gilt sowohl für die Teilnehmer als auch die Instruktoren. Idealerweise sollte dies von den Teilnehmern und Instruktoren durch Unterschrift bekräftigt werden.

iv. Simulationsbasiertes Teamtraining ist ein Ort des Lernens. Die Teilnehmer sollen informiert werden, dass auftretende Probleme bzw. „Fehler" im Szenario die Möglichkeit bieten zu lernen und es nicht darum geht, die aktiv tätigen Personen vorzuführen oder zu beurteilen.

v. Mit den Teilnehmern soll besprochen werden, dass simulationsbasierte Teamtrainings die Realität zwar sehr nahe abbilden, allerdings nicht grundsätzlich bzw. nicht immer direkte Rückschlüsse auf das Handeln bei tatsächlichen Patientenversorgungen gezogen werden können.

vi. Die Szenarien und Debriefings dürfen nur von Personen beobachtet werden, die auch an dem jeweiligen Training teilnehmen. Nur nach vorheriger Zustimmung aller Teilnehmer können Zuschauer, die nicht aktiv am Training teilnehmen, für einen vereinbarten Zeitraum zugelassen werden.

b) UMGANG MIT VIDEOS

i. Audio-Video-Aufnahmen von simulationsbasierten Teamtrainings sollen nach jedem Training gelöscht werden. Ausnahmen stellen die Verwendung für wissenschaftliche und Lehrzwecke dar. Es sind die Vorgaben der jeweiligen Ethik- und Datenschutzkommission zu beachten. Sollten Teile der Audio-Video-Aufnahmen weiter genutzt werden, muss das schriftliche Einverständnis jedes im Video sichtbaren/hörbaren Teilnehmers und ggf. der Organisationseinheit eingeholt werden.

ii. Audio-Video-Aufnahmen müssen vertraulich behandelt werden und dürfen auf keinen Fall Vorgesetzten zur Beurteilung ihrer Mitarbeiter zur Verfügung gestellt werden.

iii. Über den Umgang mit den Audio-Video-Aufnahmen müssen die Teilnehmer vor Beginn der Szenarien schriftlich informiert werden und dies per Unterschrift bestätigen.

c) UMGANG MIT FOTOS

i. Sollten Fotos während des Trainings gemacht werden, so muss ein schriftliches Einverständnis aller Teilnehmer erfolgen.

ii. Für die Verwendung von Fotos in Präsentationen, die Veröffentlichung in sozialen Medien oder die Verwendung im Internet muss das Einverständnis jedes sichtbaren Teilnehmers eingeholt werden.

iii. Fotos während der Trainingsszenarien sollten nach Möglichkeit unterbleiben, um den Szenarien-Ablauf nicht zu stören und das Erreichen von Lernzielen nicht zu gefährden.

d) ÖFFENTLICHKEITSARBEIT/ PRESSE

Sollte eine journalistische Veröffentlichung über ein Training in der Presse (extern oder intern) gewünscht sein, ist es sinnvoll, die Kollegen der Presse innerhalb der Pause über Inhalte des Trainings zu informieren und evtl. Fotos zu machen. Sollte in Ausnahmefällen Mitarbeiter der Presseabteilung für eine kurze Zeit ein Szenario beobachten, dann muss dies unbedingt mit dem Einverständnis aller Teilnehmer erfolgen.

(Anmerkung: In der Praxis hat es sich bewährt, gestellte Situationen mit Trainern zu verwenden, sofern Fotos/Videos für Öffentlichkeitsarbeit benötigt werden.)

e) LERNKLIMA

Ein wertschätzender Umgang zwischen Teilnehmern und Instruktoren ist Voraussetzung für ein positives Lernklima. Die Teilnehmer sollen ermutigt werden, Fragen zu stellen und sich aktiv an Diskussionen zu beteiligen.

Unterbrechungen und externe Störungen (z.B. Telefon, Funk) sollten vermieden werden.

f) „TOD" DES SIMULATORS

Um eine sichere Lernumgebung zu erhalten und negative Lerneffekte zu vermeiden, soll das „Sterben" des Simulators aufgrund einer Fehlbehandlung vermieden werden.[17–19]

Handelt es sich jedoch um ein ausdrückliches Lernziel (z.B. „Beenden einer Reanimation"), ist ein Sterben des Simulators im Szenario zulässig. Diese Thematik soll im Debriefing professionell aufgearbeitet und die entstehenden Emotionen aufgefangen werden.

5) SIMULATIONSUMGEBUNG

Um den Teilnehmern eine optimale Trainingsumgebung zu bieten, die es ihnen ermöglicht realitätsnah zu handeln und das Gelernte leichter in den Alltag zu übertragen, soll die Simulationsumgebung die reale Arbeitsumgebung so genau wie möglich nachbilden.[20–22]

a) RÄUMLICHKEITEN

Simulationsbasierte Teamtrainings können generell „off site" (d.h. in Simulationszentren oder in dafür vorgesehen Trainingsräumen) oder „in situ"' (d.h. am tatsächlichen Arbeitsplatz, z.B. Intensivstation, Notaufnahme, Operationssaal, Kreißsaal, Kinderarztpraxis, präklinische Einsatzorte) stattfinden, wobei grundsätzlich beide Trainingsformen in Abhängigkeit von Lernziel und Verfügbarkeit empfohlen werden können.[23]

Die folgenden Punkte können die Qualität der Trainings weiter erhöhen und die Lernumgebung optimieren, sind aber für die Durchführung von qualitativ hochwertigen Simulationstrainings nicht zwingend erforderlich:

- Simulation und Debriefing sollen bei Einsatz einer AV Anlage räumlich getrennt stattfinden. Bei „in situ"-Trainings kann für die Debriefings z.B. auf Besprechungszimmer, Elternzimmer o.Ä. zurückgegriffen werden. Bei Trainings ohne AV-Anlage kann das Debriefing auch im gleichen Raum stattfinden.
- Im Idealfall steht noch ein dritter Raum für die Steuerung zur Verfügung. Steht kein zusätzlicher Raum zur Steuerung zur Verfügung, sollte eine optische Abtrennung der Steuereinheit (z.B. durch eine mobile Wand) von dem Simulationsraum erfolgen.

b) MEDIZINISCHES EQUIPMENT IN DER SIMULATION

Empfohlen wird, dass echtes und voll funktionsfähiges medizinisches Equipment, d.h. Geräte, Medikamente, Medizinprodukte etc. verwendet werden.

Medikamente und Infusionen sollten stets tatsächlich aufgezogen und appliziert werden, um die realen Zeitverhältnisse und Probleme darstellbar zu machen.[24]

Findet die Simulation „off-site" statt, sollen die für die geplanten Szenarien notwendigen Materialien (Atemwegsequipment, Thoraxdrainagen, Perfusoren, Medikamente etc.) möglichst originalgetreu vorhanden sein.

Findet die Simulation „in-situ" statt, soll das in dem jeweiligen Arbeitsumfeld üblicherweise benutzte Equipment verwendet werden. Dabei muss darauf geachtet werden, dass die reguläre und sichere Patientenversorgung zu jedem Zeitpunkt gewährleistet und das Material in doppelter Ausführung vorhanden ist.

Bei der Verwendung von klinikeigenem Notfallmaterial muss sichergestellt werden, dass dieses nach Ende des Simulationstrainings auf Vollständigkeit und korrekte Lagerung überprüft wird.

Abgelaufene Materialien (z.B. abgelaufene Medikamente) und dezidiertes Trainingsmaterial (z.B. wiederverwendbare Intraossär-Bohrer, Beatmungsbeutel etc.) müssen als solche gekennzeichnet sein und nach dem Training unbedingt wieder vom realen medizinischen Equipment getrennt werden. Ein Rücklauf dieser Materialien in das klinische Umfeld muss unterbunden werden, um eine Verwechslung zu vermeiden. Die Verantwortlichkeit hierfür muss innerhalb des Trainerteams festgelegt sein.

Sollten sich aus dem „in situ" Training Empfehlungen zur Optimierung des realen Equipments des jeweiligen Arbeitsumfeldes ergeben, dürfen diese erst nach Absprache im gesamten lokalen Team umgesetzt werden.

c) SIMULATOREN

Für simulationsbasierte Teamtrainings sollten Patientensimulatoren mit hoher Realitätsnähe („high fidelity") oder einfachere Simulatoren („low fidelity")[25,26] mit separatem Patientenmonitor eingesetzt werden. Die Simulatoren sollten entsprechend des Lernziels gewählt werden.

In Bereichen, in denen in der Realität regelhaft kein Patientenmonitoring zur Verfügung steht, soll darauf auch in der Simulation verzichtet werden.[27]

d) STANDARDISIERTE PATIENTEN

Für geeignete Situationen (z.B. Massenanfall von Verletzten) können standardisierte Patienten (syn. Schauspieler, Statisten, Verbündete) eingesetzt werden.[4,28]

Eine weitere Möglichkeit bietet die Hybridsimulation, d.h. Einsatz von Schauspielern in Kombination mit Simulationsmaterial (z.B. Geburtssimulation).[29–31]

e) AUDIO-VIDEO ANLAGE

Eine Audio-Video-Anlage (AV-Anlage) erlaubt idealerweise die Live-Übertragung, Aufzeichnung und Wiedergabe der Szenarien mit einer oder mehreren Kameraperspektiven sowie die Aufzeichnung der Vitalparameter mit der Möglichkeit Ereignis-Marker zu setzen. Der Einsatz von AV-Anlagen bietet die Möglichkeit, dass im Szenario nicht aktiv eingebundene Teilnehmer und Trainer das Szenario mitverfolgen können, ohne im Simulationsraum anwesend zu sein. Des Weiteren kann die Selbstreflexion durch Verwendung kurzer Videosequenzen in den Debriefings verstärkt werden.[32] Die Verwendung einer AV-Anlage ist für die Effektivität eines Trainings allerdings nicht zwingend notwendig.

6) PERSONELLE RESSOURCEN

Da simulationsbasierte Teamtrainings umfangreiche zeitliche sowie personelle Ressourcen erfordern, sollte es das Ziel aller Beteiligten sein, die Simulationstrainings und die Vor- und Nachbereitung in die bezahlte Arbeitszeit zu integrieren.

a) TEILNEHMER-TEAM

i. Ideal ist eine Teamkonstellation, die der Teamzusammensetzung des realen Arbeitsalltages entspricht.[33,34] Dabei sollen die Teamtrainings interprofessionell (z.B. Pflege und Ärzte) und/oder interdisziplinär (z.B. Pädiatrie, Gynäkologie und Anästhesie) erfolgen, um Abläufe sowie Aufgabenverteilungen so real wie möglich trainieren zu können.

ii. Die Anzahl der Teilnehmer innerhalb eines Szenarios soll an Inhalt (z.B. Atemwegsproblem, Reanimation) und Setting (z.B. Intensivstation, Schockraum, Operationssaal) der Szenarien angepasst werden. Ziel ist dabei, dass die personellen Ressourcen innerhalb einer Trainingssituation der Realität entsprechend eingesetzt werden.[35,36]

iii. Im Szenario soll jeder Teilnehmer die Rolle seiner tatsächlichen persönlichen und beruflichen Position und seines Erfahrungsstands einnehmen. Der Rollenwechsel kann in Ausnahmefällen sinnvoll sein, um ein besseres Verständnis einer anderen Position zu erlangen. Dies ist dann aber ein spezifisches Lernziel des Szenarios.

iv. Die Gruppe der Teilnehmer sollte von Beginn bis Ende des Trainings, auf jeden Fall aber von Beginn bis Ende eines Szenarios inklusive Debriefing vollständig sein, um die psychologische Sicherheit und Integrität zu gewährleisten.

v. Jeder Teilnehmer soll mindestens einmal aktiv an einem Szenario als Teil des behandelnden Teams teilnehmen. Entsprechend muss die Gruppengröße des gesamten Kurses gewählt werden.

vi. Auch diejenigen Personen, die nicht aktiv an einem Szenario teilnehmen, sollen die Möglichkeit haben, das Szenario im selben Raum oder idealerweise mittels Liveübertragung in einem separaten Raum mitzuverfolgen. Dies ermöglicht, dass sich auch die Teilnehmer, welche nicht am Szenario beteiligt waren, im Debriefing einbringen und lernen können.

vii. Die Teilnehmer sollen mit den offiziellen theoretischen Richtlinien/Algorithmen passend zum jeweiligen Trainingsinhalt vertraut sein (z.B. Neugeborenen-Reanimationsalgorithmus bei neonatologischem Team-Training). Den Teilnehmern können vor Beginn des Trainings Vorbereitungsunterlagen zur Verfügung gestellt werden (z.B. Selbststudium mittels Literatur, Skript, E-Learning, Videolinks, etc.). Alternativ oder zusätzlich kann dem Simulationstraining ein Theorieteil zur Auffrischung vorangestellt werden. An dieser Stelle können theoretische Inhalte zu medizinischen und nicht-medizinischen Themen (NTS/ CRM) behandelt werden.

viii. Die Teilnehmer sollen bereits vor den simulationsbasierten Teamtrainings in technischen Fertigkeiten wie BLS, Beutel-Maskenbeatmung etc. trainiert worden sein. Dies kann sinnvollerweise in entsprechenden Trainingsformaten (siehe Anhang) geübt werden.

b) SIMULATIONSTRAINER

Ein Simulationstraining soll mit einem mindestens zweiköpfigen Trainerteam durchgeführt werden. Idealerweise wird das Zweier-Team um weitere Personen, z.B. Techniker, „SimNurse“ und/oder weiteren Trainer bzw. Debriefer, ergänzt. Alle diese Personen sollen mit den Grundsätzen der Simulation und mit dem „Ethikkodex für SimulationsexpertInnen im Gesundheitswesen“[37] (www.netzwerk-kindersimulation.org/wp-content/uploads/2019/05/Ethikkodex_German-201905.pdf) vertraut sein.

i. Debriefer

Das Debriefing sollte idealerweise von zwei Personen durchgeführt werden. Wünschenswert wären Debriefer unterschiedlicher Berufsgruppen und/oder medizinischer Disziplinen, um die jeweilige Expertise und die unterschiedlichen Sichtweisen in die Analyse einbringen zu können. Zudem bietet ein zweiter Debriefer den Vorteil, in schwierigen Situationen unterstützen zu können.[38] Im Anschluss an das Training soll ein gegenseitiges Feedback zwischen den Debriefern stattfinden, um eine kontinuierliche Weiterentwicklung zu ermöglichen. Zu Lernzwecken und Qualitätskontrolle sollten regelmäßig Debriefings

vom Debriefing durch erfahrene Instruktoren erfolgen, die nicht in das Training bzw. Debriefing involviert sind.

ii. Rollenspieler im Szenario (Statist, Schauspieler, Verbündeter)

In jedem Szenario sollte eine Person zur Verfügung stehen, die als Bindeglied zwischen Trainern und Teilnehmern fungiert (engl. „confederate“).[4,39] Die Person hält sich während des Szenarios im Simulationsraum auf und beteiligt sich nicht unaufgefordert an der Versorgung des Patienten. Sie kann anamnestische Informationen liefern bzw. Hilfestellungen geben, wenn die Teilnehmer relevante Tatsachen oder Situationsveränderungen nicht erkennen (sog. Rettungsanker, engl. „life saver“).[40]

Der Rollenspieler muss über seine vordefinierte Rolle gut informiert sein und das Ziel des Szenarios verstehen, um die Teilnehmer nicht falsch abzulenken. Bei Steuerung des Simulators von außerhalb des Simulationsraums ist es sinnvoll, dass er über ein Headset mit dem Rest der Instruktoren verbunden ist, um entsprechende Anweisungen erhalten zu können.

Die Teilnehmer sollen vor Beginn des Szenarios über die Anwesenheit des Rollenspielers informiert werden.

iii. „SimNurse“

Die Person der „SimNurse“ kann die Aufgaben des Rollenspielers übernehmen. Darüber hinaus unterstützt diese Person das Team bei der Vor- und Nachbereitung des Simulationsraums und Szenarios. Während des Szenarios hilft sie bei Fragen der Teilnehmer zu Technik oder Equipment und achtet auf die Sicherheit der Teilnehmer sowie auf den sachgerechten Umgang mit dem Simulator.

iv. Techniker

Für die Betreuung der Technik (Simulator, Mikrofone, AV-Anlage, usw.) während der Simulation sollte je nach lokalem Technikaufwand eine weitere Person zur Verfügung stehen, die alle Aspekte des vorhandenen Equipments beherrscht. Hierzu gehören Aufbau, Steuerung, Problemlösung und Abbau der Simulationsinfrastruktur.

v. Administration

Für die administrativen Arbeiten rund um das Simulationstraining (Anmeldung, Einladungsschreiben, Evaluation und Ausstellung von Zertifikaten) kann zusätzliches Personal (z.B. Sekretariatspersonal, studentischer Mitarbeiter) eingesetzt werden. Diese Personen benötigen keine spezifische Simulationsausbildung. Die Grundzüge der Simulation sollen diesen Personen jedoch bekannt sein.

7) SZENARIEN

Vordefinierte Szenarien sind ein elementarer Teil von simulationsbasierten Teamtrainings und essentiell um Lernziele zu erreichen.

a) LERNZIELE

i. Lernziele müssen klar definiert sein und in ihrem Umfang und ihrer Komplexität der Zielgruppe entsprechen.

ii. Der Umfang der Lernziele muss an die zur Verfügung stehende Trainingszeit angepasst sein.

iii. Neben medizinischem Wissen und technischen Fertigkeiten sollen Lernziele aus dem NTS (interprofessionelle Kommunikation, Rollenverteilung, Situationsbewusstsein, Entscheidungsfindung, Teamarbeit) und entsprechende Trigger mit in die Szenarien Entwicklung einbezogen werden.

iv. Der Einsatz von hilfreichen Hinweisen oder Stressoren und Ablenkungen soll dem Erreichen der Lernziele dienen und an dem Ausbildungsstand der Zielgruppe angepasst sein.

b) SZENARIENENTWICKLUNG

i. In die Entwicklung von Szenarien sollen die verschiedenen Berufsgruppen, die auch im Szenario beteiligt sind, einbezogen werden.

ii. Zum Erreichen der vordefinierten Lernziele ist es notwendig, das Szenario mit inhaltlicher, psychologischer, personeller und materieller Wiedergabetreue zu gestalten.[20,41] Zudem sollen für die Entwicklung der Szenarien die vorhandenen technischen Möglichkeiten berücksichtigt werden.

iii. Szenarien sollten vor dem ersten Einsatz mit Teilnehmern einem praktischen Test, z.B. innerhalb des Trainerteams unterzogen werden, um unvorhergesehene Probleme zu erkennen.

iv. Der Einsatz von Stressoren bzw. Ablenkungen (z.B. aufgeregte Eltern, defektes medizinisches Equipment) soll mit Bedacht erfolgen, da diese von den Lernzielen ablenken können.

v. Requisiten wie Röntgenbilder, Sonographie, Blutgasanalysen sollen dem Fall entsprechend vorbereitet werden. Hierfür können reale Fälle oder Bilder aus dem Internet verwendet werden. Die Entfernung von patientenbezogenen Daten und die Einhaltung von Copyright-Bestimmungen sind zwingend erforderlich.

vi. Szenarien sollten in regelmäßigen Abständen innerhalb des Trainerteams auf ihre Praxistauglichkeit, Relevanz und Aktualität überprüft und bei Bedarf angepasst werden.

c) SZENARIENSKRIPT

i. Die Szenarien sollen in Form von Skripten schriftlich festgelegt sein, um Reproduzierbarkeit, das Erreichen der Lernziele und Weiterentwicklung zu gewährleisten und eine Vorbereitung aller Trainer zu erlauben.

ii. Die Themenbereiche eines Skriptes sollen zumindest folgende Punkte umfassen:

- Zielgruppe
- Lernziele medizinisch und NTS
- Szenariobeschreibung für das Trainerteam: Situation, Lokalisation, beteiligte Rollenspieler im Szenario (z.B. Vater, Notarzt), Ablauf des Szenarios, Trigger für Verschlechterung oder Verbesserung, Zielsetzung
- Zusammensetzung des Teilnehmerteams
- Teilnehmer-Briefing: Wer ist im Raum? Wo findet das Szenario statt? Wann findet das Szenario statt? Was wissen wir schon vom Patienten?

- Beschreibung des geplanten Ablaufs für das Trainerteam: Vitalparameter, Trigger und Rettungsanker
- Anweisungen für Rollenspieler
- Checkliste für die Vorbereitung des Szenarios: Simulator, medizinisches Equipment, benötigte Requisiten, Dokumente, Röntgenbilder, EKG, Kleidung, Moulagen usw.[42–46]

8) ABLAUF EINES TRAININGS

a) VORBESPRECHUNG TRAINERTEAM

Vor dem Beginn einer Simulation soll innerhalb des Trainerteams eine gemeinsame Besprechung der Szenarien und Lernziele inklusive möglicher Rettungsanker sowie eine Aufgabenverteilung stattfinden. Dazu gehören Einführung in den Kurs, Familiarisierung mit dem Simulator und der Simulationsumgebung, Briefing der Teilnehmer vor den Szenarien, Steuerung der Technik, Rollenverteilung während des Szenarios und des Debriefings.

b) EINFÜHRUNG

Zu Beginn des Kurses soll zunächst eine Vorstellung von Trainern und Teilnehmern stattfinden. Anschließend müssen die Rahmenbedingungen gesetzt sowie der Ablauf des Trainings und Organisatorisches (örtliche Gegebenheiten, Zeitplan) erklärt werden. Zusätzlich können die Erwartungen der Teilnehmer erfragt werden, um im Training nach Möglichkeit darauf eingehen zu können.

c) FAMILIARISIERUNG MIT DEM SIMULATOR UND DER SIMULATIONSUMGEBUNG

Es ist wichtig, die Teilnehmer mit den Möglichkeiten (z.B. Atmung beurteilen, Intubation, Zugang legen usw.) und den Limitationen des Simulators (z.B. Hautfarbe, Bewusstsein) und der Simulationsumgebung (z.B. Notfallwagen, medizinische Geräte, Alarmierungswege, Telefone, Rollenspieler, usw.) vertraut zu machen.[16] Den Teilnehmern muss dabei mitgeteilt werden, wie sie Informationen, die der Simulator nicht wiedergeben kann, erhalten. Die Teilnehmer sollen wissen, welche personellen und materiellen Ressourcen zur Verfügung stehen und welche „scharfen" Eskalationswege (z.B. Rea-Telefonnummer) in den in-situ Settings benutzt werden können.

Die Familiarisierung soll strukturiert ablaufen (z.B. anhand des ABCDE-Schemas, des Neugeborenenalgorithmus oder mittels einer Checkliste). Die Teilnehmer sollen die Möglichkeit haben, die relevanten Simulatorfunktionen (z.B. Auskultation, Pulse, Defibrillation, Monitoring, etc.) aktiv zu testen und sich ggf. mit dem Equipment vertraut zu machen. Zusätzlich sollen, auch zum Eigenschutz, sicherheitsrelevante Aspekte (u.a. medizinische Gase, Medikamente, Defibrillationsenergie) erwähnt werden. Der Umfang der Familiarisierung kann an die Simulationserfahrung der Teilnehmer angepasst werden.

d) BRIEFING DER TEILNEHMER (VORBESPRECHUNG)

Nach Einteilung der aktiven Teilnehmer erhalten alle Teilnehmer (aktiv und passiv) eine Vorabinformation (Briefing) für das kommende Szenario. Das Briefing enthält Informationen zum Patienten, dem Setting (Notaufnahme, Intensivstation, Tageszeit usw.) und zu den im Szenario integrierten Rollenspielern (Eltern, Notarzt o.ä.).

e) DURCHFÜHRUNG DES SZENARIOS

Das Szenario soll in seinem zeitlichen Verlauf und in der Veränderung seiner Vitalparameter sofern möglich realitätsnah erfolgen. Den Teilnehmern müssen alle für das Szenario notwendigen Informationen (Anamnese, Patientenkurve, Röntgenbild, Blutgasanalyse etc.) zur Verfügung gestellt werden. Physiologische Parameter, die der Simulator nicht abbilden kann, sollen den Teilnehmern auf alternativem Weg vermittelt werden (z.B. Bewusstseinszustand durch Eltern, Rekapillarisierungszeit durch „SimNurse").

Alle notwendigen (invasiven) Maßnahmen am Simulator, (Nach-)Alarmierungen von weiterem Personal, Kommunikation mit Angehörigen etc. sollen von den Teilnehmern tatsächlich durchgeführt werden. Die Trainer müssen auf die durchgeführten Maßnahmen reagieren und den Simulator sowie die Vitalparameter zeitlich realistisch steuern (z.B. Herzfrequenzabfall nach Volumengabe bei Hypovolämie).

Sollten die Handlungen des Teams in der Realität zum „Versterben" des Simulators führen und das Versterben des Simulators kein explizites Lernziel sein, sollen Rettungsanker, z.B. Hinweise durch Rollenspieler oder Hinweise aus dem Steuerraum (engl. „Voice of God") eingesetzt oder das Szenario pausiert oder vorzeitig beendet werden.

f) DEBRIEFING (= NACHBESPRECHUNG)

Im Anschluss an das Simulationsszenario soll das Trainerteam die gesamte Gruppe direkt zum Debriefing zusammenführen, um die beginnenden Diskussionen der Teilnehmer einzufangen. Beim Debriefing soll der Fokus sowohl auf den medizinischen Inhalten als auch auf den NTS[5] und den CRM-Prinzipien liegen. Je nach vorab definierten Lernzielen, Bedarf der Teilnehmer und dem Verlauf des Szenarios kann die Gewichtung zwischen medizinischen und nicht-medizinischen Inhalten variieren.

Das Debriefing soll entsprechend erprobten Modellen, z.B. „Debriefing with good judgment"[47] oder PEARLS-Konzept[48] strukturiert durchgeführt werden.[49] Die Debriefer sollen nicht als Lehrer, sondern als Moderatoren (engl. „Facilitator") agieren.

Das Debriefing soll idealerweise aus folgenden Phasen bestehen: Übergangsphase (Abholen der Emotionen), Beschreibungsphase, Analysephase und Anwendungsphase.[50]

Die Aufrechterhaltung einer positiven und psychologisch sicheren Lernumgebung ist für ein effektives Lernen essentiell. Das bedeutet, das die Teilnehmer ein Gefühl der Sicherheit empfinden, sich nicht schämen zu müssen, abgelehnt oder anderweitig bestraft zu werden, wenn sie Fehler machen, diese eingestehen, und die wahren Gedanken mitteilen, etwas nicht zu wissen oder Fragen zu stellen.[51] Hierfür ist die Grundannahme wichtig, dass alle Teilnehmer intelligent und kompetent sind und sich ständig verbessern wollen.[52] Weiter spielen hier verschiedene implizite und explizite Strategien vor dem Debriefing (z.B. Sitzen im Kreis, Vertrag über Vertraulichkeit), während des Debriefings (z.B. Pausieren und Zuhören, vulnerabel und neugierig sein) und nach dem Debriefing (Anerkennung, Wohlwollen) eine wichtige Rolle.[51]

Die Selbstreflexion der Teilnehmer soll gefördert und Lösungsmöglichkeiten gemeinsam im Kontext erarbeitet werden. Dabei sollen geeignete Fragetechniken, z.B. 3B-Fragetechnik[53] (engl. "Advocacy and Inquiry"), zirkuläre Fragen (engl. "Circular questions"[54]), offene Fragetechnik (engl. "Open Question Technique"), angewendet werden[55].

Es sollen sich sowohl die aktiven als auch passiven Teilnehmer einbringen können. In das Debriefing können ausgewählte Videosequenzen integriert werden.[32] Diese sollen aber nicht als Videobeweis verwendet werden, sondern sollen den Teilnehmern eine strukturierte Reflexion

ermöglichen. Für diese sehr häufig verwendete Form des Debriefings sollte mindestens doppelt so viel Zeit wie für das Szenario zur Verfügung stehen.

Wir verweisen an dieser Stelle auch auf andere Modalitäten des Debriefings (z.B. „Rapid Cycle Deliberate Practice“[56] oder „3D Model of Debriefing: Defusing, Discovering, and Deepening“[57]), welche insbesondere in Nordamerika erfolgreich eingesetzt werden.

g) DURCHFÜHRUNG WEITERER SZENARIEN UND DEBRIEFINGS

Je nach Umfang des Kurses bzw. Trainings erfolgt die Durchführung weiterer Szenarien und Debriefings.

h) KURSEVALUATION

Am Ende des Kurses sollte eine schriftliche Evaluation und direktes Feedback durch die Teilnehmer erfolgen.

i) NACHBESPRECHUNG DES TRAINERTEAMS

Das Trainerteam soll sich im Anschluss an die Simulation (Szenarien, Debriefing) nachbesprechen und sich dabei untereinander konstruktives, strukturiertes Feedback bezüglich des Debriefings geben. Hilfreich können hierbei Bewertungsinstrumente wie z.B. „Objective Structured Assessment of Debriefing“ (OSAD)[58,59] oder „Debriefing Assessment for Simulation in Healthcare“ (DASH)[60] sein. Ein gegenseitiges Debriefing der Debriefings sollte regelhaft durchgeführt werden, um die Weiterentwicklung der Trainer zu unterstützen[61].

9) FEEDBACK UND EVALUATION

Feedback der Teilnehmer an die Instruktoren (entsprechend der Nachbesprechung im Trainerteam) nach dem Simulationstraining ist entscheidend für die Verbesserung des Lernerfolgs und der Qualität zukünftiger Trainings und daher zwingend einzufordern.

a) FEEDBACK DER TEILNEHMER

Eine Kursevaluation mit möglichen Verbesserungsvorschlägen erlaubt es den formalen und inhaltlichen Standard der Simulationstrainings zu überprüfen und zukünftig zu verbessern.[62,63]

Das Feedback durch die Teilnehmer soll direkt nach dem Training erfolgen, um möglichst authentische Eindrücke zu gewinnen. Es sollte idealerweise in Form eines spontanen direkten Austausches und kann zusätzlich in standardisierter anonymer schriftlicher Form erfolgen.

Zum Feedback durch die Teilnehmer gehören: Organisatorische Aspekte, Professionalität der Trainer, Darbietung der Lehrinhalte, Realitätsnähe der Szenarien, Debriefing, Lernatmosphäre, Relevanz des Erlernten für den klinischen Alltag und eine Gesamtbewertung.

b) NACHBESPRECHUNG DES TRAINERTEAMS

Im Anschluss an das Feedback durch die Teilnehmer soll eine Nachbesprechung des Trainings innerhalb des Trainerteams erfolgen. Hierbei sollen neben oben genanntem gegenseitigen Debriefing des Debriefing folgende Punkte evaluiert werden: Praktikabilität der Szenarien, technische und organisatorische Probleme, direkte Konsequenzen aus dem Teilnehmer-Feedback, Zufriedenheit der Trainer, Erreichen der Lernziele sowie mögliche Veränderungen bzw. Anpassungen der Szenarien und der Ablaufs zukünftiger Trainings besprochen werden.

c) ZUSAMMENFASSUNG DES TRAININGS

‚In Situ'-Simulationen bieten die Möglichkeit eines Systemchecks des Equipments, struktureller Abläufe (z.B. Checklisten, SOP) und von Team- und Kommunikationsaspekten.

Es hat sich bewährt, die in den Debriefings besprochenen Inhalte zur Optimierung in schriftlicher Form zu dokumentieren und den Mitarbeitern der Abteilung zur Verfügung zu stellen.

10) ANHANG

Zur Abgrenzung von simulationsbasierten Teamtrainings sind in der folgenden Tabelle die Unterschiede zu anderen Trainingsformaten dargestellt:

	Simulationsbasiertes Teamtraining	Fallbasiertes Training und Training von erweiterten Maßnahmen (ALS)	Reanimations-Basismaßnahmen (BLS)	Training technischer Fertigkeiten (Skills)
Trainer	Mindestens 2, optimal 4 Personen<br><br>2 Debriefer<br>1 Rollenspieler/ „SimNurse"<br>1 Techniker<br><br>Anforderungen: (siehe oben)<br><br>Fachkompetenz<br>Schulungskompetenz (Instruktorenstatus)<br>Kontinuierliche Fortbildungen	Mindestens 1 Person<br><br>Anforderungen:<br><br>Fachkompetenz:<br><br>Offizieller Notfallkurs des ERC (z.B. EPLS) oder AHA (z.B. PALS) und vertiefte fachliche Kompetenz im Bereich des zu vermittelnden Inhaltes<br><br>Schulungskompetenz:<br><br>Ausbildung zur Schulung der zu vermittelnden Inhalte<br><br>Regelmäßige Trainertätigkeit und eigene Fortbildung im fachlich sowie didaktischen Bereich	Mindestens 1 Person<br><br>Anforderungen:<br><br>Ausbildung zur Schulung der Basisreanimationsmaßnahmen	Mindestens 1 Person<br><br>Anforderungen:<br><br>Ausbildung zur Schulung der zu erlernenden technischen Fertigkeiten

Equipment (Mindest-anforderung)	Simulator:<br>ALS- oder „High fidelity"-Simulator mit Vitalparametermonitor oder standardisierter Patient<br>Medizinisches Equipment:<br>Echtes bzw. zumindest realitätsnahes medizinisches Equipment (obligat: Material für Atemwegsmanagement, Monitoring, Gefäßzugang [i.v./i.o.], Defibrillator, Medikamente)<br>Sonstiges:<br>Optimal: Audio-Video-System	Simulator:<br>ALS-Simulator mit Vitalparametermonitor<br>Medizinisches Equipment:<br>Echtes bzw. realitätsnahes medizinisches Equipment (zumindest Material für Atemwegsmanagement, Monitoring, Gefäßzugang [i.v./i.o.], Defibrillator, evtl. Medikamente)	Simulator:<br>Phantom mit geringer Realitätsnähe („Low fidelity")<br>Sonstiges:<br>Idealerweise Verwendung eines Feedback-Geräts [64](Qualität Ventilation bzw. Herzdruckmassage)	Simulator:<br>Phantom entsprechend der zu erlernenden Fertigkeit, z.B. Intubationstrainer, Lumbalpunktionstrainer<br>Medizinisches Equipment:<br>Reales medizinisches Equipment
Teilnehmer	Interprofessionelle und ggf. interdisziplinäre Teams in realistischer Team-Konstellation<br>Nach Möglichkeit mit BLS bzw. ALS Vorkenntnissen	Beliebig zusammengesetzte Teilnehmergruppen<br>Idealerweise interprofessionell	Beliebig zusammengesetzte Teilnehmergruppen	Beliebig zusammengesetzte Teilnehmergruppen
Ort / Setting	Simulationszentrum „off-site" oder „in situ"<br>Optimal:<br>1 Trainingsraum<br>1 Debriefingraum<br>1 Technikraum/Steuerraum	1 Trainingsraum<br>Optional:<br>1 Debriefingraum	1 Trainingsraum	1 Trainingsraum

Nachbesprechung / Debriefing	Strukturierte Nachbesprechung der medizinischen und nicht technischen Fertigkeiten auf der individuellen, Team- und systemischen Ebene	Fokus auf medizinischen Inhalten und Abläufen inklusive einer Analyse der Umsetzung dieser Maßnahmen im Team	Fokus auf technische Fertigkeiten und Algorithmus	Fokus auf die zu erlernenden technischen Fertigkeiten
Lernziele	Medizinische Inhalte sowie nicht-technische Aspekte im interprofessionellen und/oder interdisziplinären Team<br>Anwendung von Algorithmen und erlernten Fertigkeiten in komplexen Situationen<br>Umsetzung der Maßnahmen im Team	Erkennen und Einschätzen eines kritisch kranken Kindes<br>Anwendung von Algorithmen und erlernten Fertigkeiten in komplexen Situationen	Beherrschen des BLS Algorithmus mit effektiver Beutel-Maskenbeatmung und Herzdruckmassage	Technische Fertigkeiten beherrschen

Tabelle 1: Übersicht unterschiedlicher simulationsbasierter Trainingsformate

11) LITERATUR

1. Deutsche Gesellschaft für Simulation in der Medizin e. V. (DGSiM e.V.), Mindestanforderungen für Teamtrainings der DGSiM. http://www.dgsim.de/sites/default/files/dgsim_mindestanforderungen_teamtraining_version_1.pdf (2015).

2. Society in Europe for Simulation applied to medicine (SESAM). SESAM Accreditation of Simulation Based Educational Institutions – Principles. https://worldspanmedia.s3.amazonaws.com/media/sesam2/uploads/documents/accreditation/sesam-accreditation-of-simulation-based-educational-institutions-principles-2018.pdf.

3. Sittner, B. J. *et al.* INACSL Standards of Best Practice for simulation: Past, present, and future. *Nurs. Educ. Perspect.* (2015) doi:10.5480/15-1670.

4. Lioce L. (Ed.), Downing D., Chang T.P., Robertson J.M., Anderson M., Diaz D.A., and S. A. E. (Assoc. E. . and the T. and C. W. G. (2020). Healthcare Simulation Dictionary –Second Edition. *Rockville, MD: Agency for Healthcare Research and Quality* AHRQ Publication No. 20-0019 https://www.ahrq.gov/patient-safety/resources/simulation/terms.html.

5. Rhona, F., O'Connor, P. & Crichton, M. D. *Safety at the sharp end: a guide to non-technical skills.* (2008).

6. Rall, M. & Lackner, C. K. Crisis Resource Management (CRM)Crisis resource management (CRM). *Notfall + Rettungsmedizin* (2010) doi:10.1007/s10049-009-1271-5.

7. Kanki, B. G. Communication and Crew Resource Management. in *Crew Resource Management* (2010). doi:10.1016/B978-0-12-374946-8.10004-4.

8. Eppich, W. 'Speaking Up' for Patient Safety in the Pediatric Emergency Department. *Clin. Pediatr. Emerg. Med.* (2015) doi:10.1016/j.cpem.2015.04.010.

9. Schmutz, J. B., Lei, Z., Eppich, W. J. & Manser, T. Reflection in the heat of the moment: The role of in-action team reflexivity in health care emergency teams. *J. Organ. Behav.* (2018) doi:10.1002/job.2299.

10. Schmutz, J. B., Kolbe, M. & Eppich, W. J. Twelve tips for integrating team reflexivity into your simulation-based team training. *Med. Teach.* (2018) doi:10.1080/0142159X.2018.1464135.

11. Salas, E., Sims, D. E. & Shawn Burke, C. Is there A 'big five' in teamwork? *Small Group Research* (2005) doi:10.1177/1046496405277134.

12. El-Shafy, I. A. *et al.* Closed-Loop Communication Improves Task Completion in Pediatric Trauma Resuscitation. *J. Surg. Educ.* (2018) doi:10.1016/j.jsurg.2017.06.025.

13. Topping, A. *et al.* Towards identifying nurse educator competencies required for simulation-based learning: A systemised rapid review and synthesis. *Nurse Education Today* (2015) doi:10.1016/j.nedt.2015.06.003.

14. Roh, Y. S. & Jang, K. I. Survey of factors influencing learner engagement with simulation debriefing among nursing students. *Nurs. Heal. Sci.* (2017) doi:10.1111/nhs.12371.

15. Edmondson, A. Psychological safety and learning behavior in work teams. *Adm. Sci. Q.* (1999) doi:10.2307/2666999.

16. Rudolph, J. W., Raemer, D. B. & Simon, R. Establishing a safe container for learning in simulation the role of the presimulation briefing. *Simulation in Healthcare* (2014) doi:10.1097/SIH.0000000000000047.

17. Calhoun, A. W. & Gaba, D. M. Live or Let Die. *Simulation in Healthcare* (2017) doi:10.1097/SIH.0000000000000256.

18. McBride, M. E., Schinasi, D. A., Moga, M. A., Tripathy, S. & Calhoun, A. Death of a simulated pediatric patient: Toward a more robust theoretical framework. *Simul. Healthc.* (2017) doi:10.1097/SIH.0000000000000265.

19. Tripathy, S. *et al.* When the Mannequin Dies, Creation and Exploration of a Theoretical Framework Using a Mixed Methods: Approach. *Simul. Healthc.* (2016) doi:10.1097/SIH.0000000000000138.

20. Dieckmann, P., Gaba, D. & Rall, M. Deepening the theoretical foundations of patient simulation as social practice. *Simulation in Healthcare* (2007) doi:10.1097/SIH.0b013e3180f637f5.

21. Nanji, K. C., Baca, K. & Raemer, D. B. The effect of an olfactory and visual cue on realism and engagement in a health care simulation experience. *Simul. Healthc.* (2013) doi:10.1097/SIH.0b013e31827d27f9.

22. Appendix 1. https://www.faa.gov/about/initiatives/nsp/ac/media/AC_120-40B-Ch2.pdf.

23. Sørensen, J. L. *et al.* Design of simulation-based medical education and advantages and disadvantages of in situ simulation versus off-site simulation. *BMC Med. Educ.* (2017) doi:10.1186/s12909-016-0838-3.

24. Meurling, L. *et al.* Comparison of high- and low equipment fidelity during paediatric simulation team training: A case control study. *BMC Med. Educ.* (2014) doi:10.1186/1472-6920-14-221.

25. Gu, Y. *et al.* The effect of simulator fidelity on acquiring non-technical skills: a randomized non-inferiority trial. *Can. J. Anesth.* (2017) doi:10.1007/s12630-017-0973-2.

26. Finan, E., Bismilla, Z., Whyte, H. E., LeBlanc, V. & McNamara, P. J. High-fidelity simulator technology may not be superior to traditional low-fidelity equipment for neonatal resuscitation training. *J. Perinatol.* (2012) doi:10.1038/jp.2011.96.

27. Langdorf, M. I. *et al.* High-Fidelity Simulation Enhances ACLS Training. *Teach. Learn. Med.* (2014) doi:10.1080/10401334.2014.910466.

28. Cantrell, M. J. & Deloney, L. A. Integration of Standardized Patients into Simulation. *Anesthesiology Clinics* (2007) doi:10.1016/j.anclin.2007.03.003.

29. Bullough, A. S. *et al.* Obstetric team simulation program challenges. *J. Clin. Anesth.* (2016) doi:10.1016/j.jclinane.2016.08.019.

30. Unver, V. *et al.* Integrating simulation based learning into nursing education programs: Hybrid simulation. *Technol. Heal. Care* (2018) doi:10.3233/THC-170853.

31. Ghi, T. *et al.* The use of a hybrid mannequin for the modern high-fidelity simulation in the labor ward: the Italian experience of the Ecografia Gestione Emergenze Ostetriche (EGEO) group. *Am. J. Obstet. Gynecol.* (2019) doi:10.1016/j.ajog.2019.07.023.

32. Sawyer, T. *et al.* The effectiveness of video-assisted debriefing versus oral debriefing alone at improving neonatal resuscitation performance a randomized trial. *Simulation in Healthcare* (2012) doi:10.1097/SIH.0b013e3182578eae.

33. Ker, J., Mole, L. & Bradley, P. Early introduction to interprofessional learning: A simulated ward environment. *Med. Educ.* (2003) doi:10.1046/j.1365-2923.2003.01439.x.

34. Vanderbilt, A. A., Pappada, S. M., Stein, H., Harper, D. & Papadimos, T. J. Increasing patient safety with neonates via handoff communication during delivery: a call for interprofessional health care team training across GME and CME. *Adv. Med. Educ. Pract.* (2017) doi:10.2147/amep.s129674.

35. Lim, Y. S., Steinemann, S. & Berg, B. W. Team size impact on assessment of teamwork in simulation-based trauma team training. *Hawaii. J. Med. Public Health* (2014).

36. Kelleher, D. C., Kovler, M. L., Waterhouse, L. J., Carter, E. A. & Burd, R. S. Factors affecting team size and task performance in pediatric trauma resuscitation. *Pediatr. Emerg. Care* (2014) doi:10.1097/PEC.0000000000000106.

37. Ethikkodex. https://www.netzwerk-kindersimulation.org/wp-content/uploads/2019/05/Ethikkodex_German-201905.pdf.

38. Cheng, A. *et al.* Co-debriefing for simulation-based education: A primer for facilitators. *Simulation in Healthcare* (2015) doi:10.1097/SIH.0000000000000077.

39. Sanko, J. S., Shekhter, I., Kyle, R. R., Di Benedetto, S. & Birnbach, D. J. Establishing a convention for acting in healthcare simulation: Merging art and science. *Simulation in Healthcare* (2013) doi:10.1097/SIH.0b013e318293b814.

40. Dieckmann, P., Lippert, A., Glavin, R. & Rall, M. When things do not go as expected: Scenario life savers. *Simul. Healthc.* (2010) doi:10.1097/SIH.0b013e3181e77f74.

41. Rudolph, J. W., Simon, R. & Raemer, D. B. Which reality matters? Questions on the path to high engagement in healthcare simulation. *Simulation in Healthcare* (2007) doi:10.1097/SIH.0b013e31813d1035.

42. Seropian, M. A. General Concepts in Full Scale Simulation: Getting Started. *Anesthesia and Analgesia* (2003) doi:10.1213/01.ANE.0000090152.91261.D9.

43. Alinier, G. Developing high-fidelity health care simulation scenarios: A guide for educators and professionals. *Simul. Gaming* (2011) doi:10.1177/1046878109355683.

44. Benishek, L. E. *et al.* The Template of Events for Applied and Critical Healthcare Simulation (TEACH Sim). *Simul. Healthc. J. Soc. Simul. Healthc.* (2015) doi:10.1097/sih.0000000000000058.

45. Lioce, L. *et al.* Standards of Best Practice: Simulation Standard IX: Simulation Design. *Clin. Simul. Nurs.* (2015) doi:10.1016/j.ecns.2015.03.005.

46. Huffman, J. L., McNeil, G., Bismilla, Z. & Lai, A. Essentials of Scenario Building for Simulation-Based Education. in (2016). doi:10.1007/978-3-319-24187-6_2.

47. Rudolph, J. W., Simon, R., Rivard, P., Dufresne, R. L. & Raemer, D. B. Debriefing with Good Judgment: Combining Rigorous Feedback with Genuine Inquiry. *Anesthesiology Clinics* (2007) doi:10.1016/j.anclin.2007.03.007.

48. Eppich, W. & Cheng, A. Promoting excellence and reflective learning in simulation (PEARLS): Development and rationale for a blended approach to health care simulation debriefing. *Simul. Healthc.* **10**, 106–115 (2015).

49. Sawyer, T., Eppich, W., Brett-Fleegler, M., Grant, V. & Cheng, A. More Than One Way to Debrief. *Simul. Healthc. J. Soc. Simul. Healthc.* (2016) doi:10.1097/sih.0000000000000148.

50. Eppich, W. & Cheng, A. Promoting Excellence and Reflective Learning in Simulation (PEARLS). *Simul. Healthc. J. Soc. Simul. Healthc.* (2015) doi:10.1097/sih.0000000000000072.

51. Kolbe, M. *et al.* Managing psychological safety in debriefings: A dynamic balancing act. *BMJ Simul. Technol. Enhanc. Learn.* (2019) doi:10.1136/bmjstel-2019-000470.

52. Rudolph, J. W., Simon, R., Raemer, D. B. & Eppich, W. J. Debriefing as formative assessment: Closing performance gaps in medical education. in *Academic Emergency Medicine* (2008). doi:10.1111/j.1553-2712.2008.00248.x.

53. Rudolph, J. W., Simon, R., Dufresne, R. L. & Raemer, D. B. There's no such thing as 'nonjudgmental' debriefing: a theory and method for debriefing with good judgment. *Simul. Healthc.* (2006) doi:10.1097/01266021-200600110-00006.

54. Kolbe, M., Marty, A., Seelandt, J. & Grande, B. How to debrief teamwork interactions: using

circular questions to explore and change team interaction patterns. *Adv. Simul.* (2016) doi:10.1186/s41077-016-0029-7.

55. Dieckmann, P., Friis, S. M., Lippert, A. & Østergaard, D. The art and science of debriefing in simulation: Ideal and practice. *Med. Teach.* (2009) doi:10.1080/01421590902866218.

56. Hunt, E. A. *et al.* Pediatric resident resuscitation skills improve after 'Rapid Cycle Deliberate Practice' training. *Resuscitation* (2014) doi:10.1016/j.resuscitation.2014.02.025.

57. Zigmont, J. J., Kappus, L. J. & Sudikoff, S. N. The 3D Model of Debriefing: Defusing, Discovering, and Deepening. *Seminars in Perinatology* (2011) doi:10.1053/j.semperi.2011.01.003.

58. Runnacles, J., Thomas, L., Sevdalis, N., Kneebone, R. & Arora, S. Development of a tool to improve performance debriefing and learning: The paediatric Objective Structured Assessment of Debriefing (OSAD) tool. *Postgrad. Med. J.* (2014) doi:10.1136/postgradmedj-2012-131676.

59. Runnacles, J., Thomas, L., Korndorffer, J., Arora, S. & Sevdalis, N. Validation evidence of the paediatric Objective Structured Assessment of Debriefing (OSAD) tool. *BMJ Simul. Technol. Enhanc. Learn.* (2016) doi:10.1136/bmjstel-2015-000017.

60. DASH. https://harvardmedsim.org/debriefing-assessment-for-simulation-in-healthcare-dash-german/.

61. Cheng, A. *et al.* Coaching the Debriefer. *Simul. Healthc.* (2017) doi:10.1097/SIH.0000000000000232.

62. McOwen, K. S., Bellini, L. M., Morrison, G. & Shea, J. A. The development and implementation of a health-system-wide evaluation system for education activities: Build it and they will come. *Academic Medicine* (2009) doi:10.1097/ACM.0b013e3181b6c996.

63. Schiekirka, S. *et al.* Student perceptions of evaluation in undergraduate medical education: A qualitative study from one medical school. *BMC Med. Educ.* (2012) doi:10.1186/1472-6920-12-45.

64. Wagner, M. *et al.* Effects of feedback on chest compression quality: A randomized simulation study. *Pediatrics* **143**, (2019).
