# Supplementary material for: Recommendations of the Netzwerk Kindersimulation for the Implementation of Simulation-Based Pediatric Team Trainings: A Delphi Process
Source: Children (Basel). 2023 Jun 16;10(6):1068. doi: 10.3390/children10061068 (PMC10297481; doi:10.3390/children10061068)
Supplement: Supplementary file 1 [file children-10-01068-s001.zip › children-2415882-supplementary/Quality criteria/Supplementary Materials File S2. Quality Criteria_First Edition_June 2020.pdf]

Recommendations

of the Netzwerk Kindersimulation e.V. [Paediatric Simulation Network Inc.] for the implementation of simulation-based paediatric team trainings

Ruth M. Löllgen, Lukas P. Mileder, Katharina Bibl, Christian Dörfler, Annika Paulun, Jasmin Rupp, Benedikt Sandmeyer, Alex Staffler, Michael Wagner, Ellen Heimberg

First Edition June 2020

NETZWERK
KINDERSIMULATION

Contakt

vorstand@netzwerk-kindersimulation.org

Universitätsklinikum Tübingen (University Hospital)
Children's Hospital
Hoppe-Seyler-Str. 1
72076 Tübingen
Deutschland

www.netzwerk-kindersimulation.org

NETZWERK
KINDERSIMULATION

List of Abbreviations

AHA	American Heart Association
ALS	Advanced Life Support
BLS	Basic Life Support
CRM	Crisis Resource Management/ Crew Resource Management
DASH	Debriefing Assessment for Simulation in Healthcare
EPALS	European Pediatric Advanced Life Support
ERC	European Resuscitation Council
HRO	High Reliability Organisation
NLS	Newborn Life Support
NRP	Neonatal Resuscitation Program
NTS	Non-Technical Skills
OSAD	Objective Structured Assessment of Debriefing
PALS	Pediatric Advanced Life Support
SOP	Standard Operating Procedure
OSCE	Objective Structured Clinical Examination
CIRS	Critical Incident Reporting System

**1)**	**PREAMBLE**	**3**
**2)**	**GENERAL LEARNING OBJECTIVES OF A SIMULATION-BASED TEAM TRAINING**	**4**
**3)**	**SKILLS & QUALIFICATIONS OF SIMULATION TRAINERS**	**4**
a)	PROFESSIONAL MEDICAL PREREQUISITES	4
b)	EMERGENCY TRAINING	4
c)	SIMULATION TRAINER TRAINING	5
d)	NON-MEDICAL SKILLS ("NON-TECHNICAL SKILLS")	5
e)	SIMULATION MENTORING	5
f)	CONTINUING PROFESSIONAL DEVELOPMENT IN SIMULATION	5
g)	MINIMUM REQUIREMENT FOR THE NUMBER OF SIMULATION TRAININGS	6
**4)**	**FRAMEWORK CONDITIONS FOR AN EFFECTIVE LEARNING ENVIRONMENT**	**6**
a)	CONFIDENTIALITY	6
b)	HANDLING OF VIDEOS	6
c)	HANDLING OF FOTOS	7
d)	PUBLIC RELATIONS / PRESS	7
e)	LEARNING ENVIRONMENT	7
f)	"DEATH" OF THE SIMULATOR	7
**5)**	**SIMULATION ENVIRONMENT**	**8**
a)	PREMISES	8
b)	MEDICAL EQUIPMENT IN SIMULATION	8
c)	SIMULATORS	9
d)	STANDARDISED PATIENTS	9
e)	AUDIO-VISUAL SYSTEM	9
**6)**	**HUMAN RESOURCES**	**9**
a)	PARTICIPANT TEAM	9
b)	SIMULATION TRAINERS	10
**7)**	**SCENARIOS**	**11**
a)	LEARNING OBJECTIVES	11
b)	SCENARIO DEVELOPMENT	11
c)	SCENARIO SCRIPT	12
**8)**	**PROCESS SEQUENCE OF A TRAINING**	**12**
a)	PREBRIEFING OF TRAINER TEAM	12
b)	INTRODUCTION	12
c)	FAMILIARISATION WITH THE SIMULATOR AND SIMULATION ENVIRONMENT	13
d)	BRIEFING OF PARTICIPANTS (PREBRIEFING)	13
e)	IMPLEMENTING THE SCENARIO	13
f)	DEBRIEFING	13
g)	IMPLEMENTATION OF OTHER SCENARIOS AND DEBRIEFINGS	14
h)	COURSE EVALUATION	14
i)	DEBRIEFING OF TRAINER TEAM	14
**9)**	**FEEDBACK AND EVALUATION**	**15**
a)	FEEDBACK BY PARTICIPANTS	15
b)	DEBRIEFING OF TRAINER TEAM	15
c)	SUMMARY OF THE TRAINING	15
**10)**	**APPENDIX**	**16**
**11)**	**LITERATURE**	**18**

1) PREAMBLE

Netzwerk Kindersimulation e.V. [Paediatric Simulation Network Inc.] has developed the following recommendations to ensure a uniform standard for the implementation of simulation-based team trainings for paediatric emergencies and a high level of quality in simulation-based education, training and continuing education in German-speaking countries. These expert recommendations have been accredited by the German Society for Neonatology and Paediatric Intensive Care Medicine (GNPI).

The recommendations are guided, where applicable, by the current scientific literature as well as by existing recommendations / quality criteria of relevant specialist societies.[1–3] Due to paucity of scientific evidence in many areas of simulation-based education and training, numerous multidisciplinary experts were involved in the preparation of these multidisciplinary recommendations in order to incorporate existing knowledge and experience in the design and implementation of paediatric simulation training in an optimal manner. The primary focus of this document is on simulation-based team training, which explicitly distinguishes these recommendations from those for other forms of training (see Table 1). However, the table in the appendix contains summarised recommendations for minimum standards in all fields of simulation for the purpose of a better overview or comparability.

The present recommendations are addressed to all persons who are, or aspire to be, involved in the health care system in the preclinical domain, outpatient care as well as in-hospital (labour ward / delivery suite, paediatric and neonatal intensive care units, emergency departments, operating room, recovery room, day clinics, paediatric wards, etc.) in paediatric education, further education and training by means of simulation-based team training. Furthermore, these recommendations are also directed at all those persons engaged in a managerial or supervisory capacity in the field of simulation and/or as trainers. In general, the recommendations summarised here are independent of the team training location and refer both to training in a simulation centre and to training "in situ", i.e. at the actual workplace. The recommendations do not distinguish between the different degrees of reality of the simulators and simulation objects used.

These recommendations describe the material equipment and staffing required for simulation training as well as the contents and structure of simulation-based team training. The aim is to achieve the highest possible quality of training by standardising these training courses, thus improving the care of sick and injured newborns and children and increasing patient safety.

The following recommendations are intended to serve as a framework for the organisation, implementation and quality assurance of these training courses. Specific recommendations on technical equipment and detailed contents, e.g. scenarios and debriefing techniques, do not form part of the recommendations. In general, to maintain the quality of simulation programs, at least one responsible person should be designated who regularly checks compliance with these recommendations and initiates corrective measures, if necessary.

In the text, a gradation of recommendations is applied according to expert opinion and, if applicable, according to evidence. This recommendation gradation is divided into "shall", "should" and "may" in descending order of recommendation strength.

Throughout the text, the masculine gender form is purposely used for ease of reading. Unless explicitly stated, person-specific information always refers to all genders in equal measure.

The definition of any specialist terms in the text is based on the contents of the Healthcare Simulation Dictionary of the Society for Simulation in Healthcare™ (https://www.ssih.org/dictionary)[4].

The following recommendations refer to traditional simulation-based team training and explicitly do not specifically cover the field of tele-simulation, e-simulation, remote simulation, or virtual simulation, although many recommendations may overlap.

2) GENERAL LEARNING OBJECTIVES OF SIMULATION-BASED TEAM TRAINING

The primary goal of simulation training is the improvement of patient safety.

This is achieved through high-quality training adapted to the level of experience of the participants, which includes the enhancement of medical-technical, cognitive and social skills as well as the approach to dealing with complex medical situations.

It should be ensured that, in addition to communication aspects, participants learn the following principles[5]:

- Situational awareness
- Task management
- Team work
- Decision making

3) SKILLS & QUALIFICATIONS OF SIMULATION TRAINERS

The selection of simulation trainers is of paramount importance, as their task places high demands on professional and social competence. The following qualifications shall apply to at least one of the trainers present at a training course. The second trainer may still be undergoing further training in all these skills.

a) PROFESSIONAL MEDICAL PREREQUISITES

The minimum requirement shall be three years of practical experience (full-time or part-time equivalent) in paediatric and adolescent medicine (paediatrics, paediatric surgery) or three years of experience in the medical care of newborns and children (paediatric nursing, midwifery, anaesthesia, paramedic/ambulance training). Special expertise in the fields of neonatology, paediatric intensive care and/or paediatric emergency medicine is advantageous.

b) EMERGENCY TRAINING

Successful participation in a certified paediatric emergency course (e.g. European Pediatric Advanced Life Support (EPALS) by the European Resuscitation Council (ERC) or Pediatric Advanced Life Support PALS by the American Heart Association (AHA) for paediatric emergency training courses; or Newborn Life Support (NLS) by the ERC; or Neonatal Resuscitation Program (NRP) by the AHA for neonatal emergency training courses) or comparable in-hospital certified course formats for advanced emergency procedures performed on children, as well as maintaining the provider status through regular participation in appropriate refresher courses, is a fundamental requirement.

It must be ensured that scenario contents and debriefings correspond to the currently published guidelines and that these are promptly adjusted when the guidelines are updated.

c) SIMULATION TRAINER TRAINING

Simulation trainers shall complete a train-the-trainer course for simulation which contains the following contents: Crisis Resource Management (CRM[6,7] = principles of individual and especially joint action in routine and crisis situations that enable effective decision-making and cooperation), tools for the improvement of patient safety, scenario design, theoretical contents on debriefings and the practical implementation of debriefings. In this context, scenario development, preparation and implementation as well as debriefing shall each have been actively conducted at least once and relevant feedback shall have been received from the course trainers.

d) NON-MEDICAL SKILLS ("NON-TECHNICAL SKILLS")

Simulation trainers shall be proficient in non-medical skills ("Non-Technical Skills", NTS). These include, for example: 15 principles of CRM [6,7], "Speaking-Up"[8], allocation of tasks in teams (team leaders and team members), implementation of team reflections [9,10] (STOP sequences, "10 seconds for 10 minutes"[6]), effective communication strategies[11,12] (e.g. "closed-loop" communication), correct use of checklists, development of common mental models.[4,5]

Furthermore, fundamental aspects of the following themes should be covered or learned:

Tools to improve patient safety, standardisation ("Standard Operating Procedures", SOP), critical incident reporting systems as well as systemic safety in High Reliability Organisations (HRO).

e) SIMULATION MENTORING

After completion of a train-the-trainer course, an experienced mentor should supervise the consolidation of the following areas. If this option is not available in a particular setting, experts from Netzwerk Kindersimulation may be engaged for the purpose of work shadowing. Published evidence regarding the duration, frequency and process of such mentoring is still scarce, but based on our own unpublished experience we recommend regular joint simulation training sessions with an experienced simulation trainer and (de)briefing of the following contents:

- Selection and preparation of scenarios
- Introduction to simulation training
- Briefing of extras or confederates[4]: Actors, standardised patients = people other than the patient who participate in a scripted simulation to provide added realism or additional information for the learners)
- Familiarisation
- Simulator operation and technical equipment
- Conducting a simulation training
- Co-Debriefing
- Debriefing

f) CONTINUING PROFESSIONAL DEVELOPMENT IN SIMULATION

In order to maintain and further develop their knowledge, simulation trainers shall undergo at least four hours of further training on simulation and/or debriefing (internal or external trainer-peer-feedback, telesimulation, shadowing in other institutions, workshops, train-the-trainer

courses, simulation-specific congresses / training courses, etc.) at least once a year. Membership in the Paediatric Simulation Network is desirable.

g) MINIMUM REQUIREMENT FOR THE NUMBER OF SIMULATION TRAININGS

To maintain the quality of teaching, scenarios including debriefings shall be conducted regularly. A minimum of 10 scenarios + debriefings per year should be aimed for.

4) FRAMEWORK CONDITIONS FOR AN EFFECTIVE LEARNING ENVIRONMENT

To enable participants to learn effectively, a safe learning environment is essential.[13–15] Therefore, a "protected space" must be provided when conducting simulation-based team trainings and debriefings, which allows participants to feel safe. To ensure this "psychological safety" for the participants, the following basic rules must be observed.[16]

a) CONFIDENTIALITY

i. During the introduction of the course, but most definitely before commencing the scenarios, the most important rules must be discussed with the participants. Where appropriate, these may also be communicated in writing before the start of the course.

ii. The participants must be assured that information about the performance of participants will not be communicated to anyone outside the simulation training (especially not to superiors, administration and/or clinic management). The only contents that shall be communicated are problems of a systemic nature, e.g. technical, logistical or equipment problems. Individual persons involved must not be identified when communicating these contents. An exception (but not scope of these recommendations) are e.g. simulation-based assessments of students or graduates of a specialist doctor, specialist nursing, paramedic, midwife examination etc., in which the performance of the participants is transparently communicated to the examination board, e.g. within the framework of an OSCE ("Objective Structured Clinical Examination" [4]).

iii. The contents of the scenarios and debriefings must be kept confidential. This applies equally to the participants and the instructors. Ideally, this should be confirmed by the participants and instructors with their signatures.

iv. Simulation-based team training is a place of learning. Participants shall be informed that any problems or "errors" that occur in the scenario provide an opportunity to learn and that the objective is not to expose, shame or judge the people actively involved.

v. It shall be discussed with the participants that although simulation-based team trainings reflect reality very closely, it is not always possible to draw direct conclusions about how to perform actual patient care.

vi. The scenarios and debriefings may only be witnessed by persons who also participate in the respective training. Observers who are not actively participating in the training may be admitted for an agreed period only with the prior consent of all participants.

b) HANDLING OF VIDEOS

i. Audio-visual recordings of simulation-based team trainings shall be deleted after each training. Exceptions are the use for scientific and teaching purposes. The guidelines of the respective ethics and data protection committee must be observed. If any parts of the audio-

visual recordings are to be used for other purposes, the written consent of each participant visible/audible in the video and, if applicable, of the organisational unit must be obtained.

ii. Audio-visual recordings must be treated confidentially and may under no circumstances be made available to superiors for the purpose of evaluating their employees.

iii. The participants must be informed in writing about the handling of the audio-visual recordings before the commencement of the scenarios and confirm this with their signature.

c) HANDLING OF PHOTOGRAPHS

i. If photos are to be taken during the training, the written consent of all participants must be obtained.

ii. The consent of every visible participant must be obtained for the use of photos in presentations, publication in social media or use on the Internet.

iii. The taking of photos during the training scenarios should be avoided if possible, in order not to disrupt the scenario process and not to jeopardise the achievement of learning objectives.

d) PUBLIC RELATIONS / PRESS

If a journalistic publication about the training in the press (external or internal) is desired, it is advisable to inform the members of the press about the contents of the training during the break and to take photos at that time if necessary. If, in exceptional cases, members of the press department observe a scenario for a brief period of time, it is essential to do so only with the consent of all participants.

(Note: Practice has consistently shown that it is best to use staged scenes with trainers when photos or videos are needed for public relations purposes).

e) LEARNING ENVIRONMENT

A respectful interaction between participants and instructors is a prerequisite for a positive learning environment. Participants should be encouraged to ask questions and actively participate in discussions.

Disruptions and external interference (e.g. telephone, radio) should be avoided.

f) "DEATH" OF THE SIMULATOR

In order to maintain a safe learning environment and to avoid negative learning effects, "dying" of the simulator due to incorrect treatment shall be avoided.[17–19]

However, if it is an explicit learning objective (e.g. "terminating a resuscitation"), dying of the simulator in the scenario is allowed. This issue shall be professionally worked through in the debriefing and the resulting emotions shall be cushioned.

5) SIMULATION ENVIRONMENT

In order to offer the participants an optimal training environment which enables them to act under realistic conditions and to transfer what they have learned more easily into everyday life, the simulation environment shall replicate the real working environment as faithfully as possible.[20–22]

a) PREMISES

Simulation-based team training may generally take place "off site" (i.e. in simulation centres or in training rooms provided for this purpose) or "in situ" (i.e. at the actual workplace, e.g. intensive care unit, emergency department, operating theatre, delivery room, paediatrician's practice, pre-clinical operational sites), but in principle both forms of training may be recommended depending on the learning objective and availability.[23]

The following aspects may further increase the quality of the training and optimise the learning environment, but are not absolutely necessary for the delivery of high quality simulation training:

- Simulation and debriefing shall take place in separate rooms when using an AV system. In the case of "in situ" training courses, the debriefings may be held in a meeting room, parents' room or similar. For training sessions without AV equipment, the debriefing may also take place in the same room.
- In the ideal case, a third room would be available for operating the equipment. If there is no additional space available for operating the equipment, a visual separation of the control unit (e.g. by means of a partition wall) from the simulation room should be provided.

b) MEDICAL EQUIPMENT IN SIMULATION

It is recommended that genuine and fully functional medical equipment, i.e. devices, medication, medical products, etc. is used.

Medicines and infusions should always actually be drawn up and applied in order to depict the real time conditions and problems.[24]

If the simulation takes place "off-site", the materials required for the planned scenarios (respiratory equipment, thoracic drainages, perfusors, medication, etc.) shall be available in the most authentic form possible.

If the simulation takes place "in-situ", the equipment normally used in the respective working environment shall be used. At the same time, care must be taken to ensure that regular and safe patient care is guaranteed at all times and that the material is available in duplicate.

When using the hospital's own emergency materials, it must be ensured that these are checked for completeness and correct storage after the end of the simulation training.

Expired materials (e.g. out-of-date medication) and dedicated training materials (e.g. reusable intra-osseous drills, resuscitator bags, etc.) must be clearly marked as such and must be separated from the real medical equipment after the training. A recirculation of these materials into the clinical environment must be avoided in order to prevent mix-ups. The responsibility for this must be determined within the trainer team.

Should the "in situ" training result in recommendations for optimising the real equipment of the respective working environment, these may only be implemented after consultation with the entire local team.

c) SIMULATORS

For simulation-based team training, high-fidelity patient simulators or simpler simulators ("low-fidelity")[25,26] with separate patient monitors should be used. The simulators should be chosen in accordance with the learning objective.

In areas where in reality no patient monitoring is usually available, it shall also be dispensed with in the simulation.[27]

d) STANDARDISED PATIENTS

For suitable situations (e.g. mass casualties) standardised patients (syn. actors, extras, confederates) may be assigned.[4,28]

Another opportunity is available with hybrid simulation, i.e. the use of actors in combination with simulation material (e.g. birth simulation).[29–31]

e) AUDIO-VISUAL SYSTEM

An audio-visual (AV) system ideally permits the live transmission, recording and playback of scenarios with one or more camera angles as well as the recording of vital parameters with the possibility of setting event markers. The use of AV equipment makes it possible for participants and trainers not actively involved in the scenario to follow the scenario without being physically present in the simulation room. Furthermore, self-reflection can be enhanced by using short video sequences in the debriefings.[32] However, the use of an AV system is not absolutely necessary for the effectiveness of a training.

6) HUMAN RESOURCES

As simulation-based team trainings require extensive time as well as human resources, it should be the goal of all stakeholders to integrate the simulation trainings as well as the preparation and wrap-up into the paid work schedule.

a) PARTICIPANT TEAM

i. The ideal team constellation is one that mirrors the team composition of the real working day.[33,34] The team training shall be interprofessional (e.g. nurses and doctors) and/or interdisciplinary (e.g. paediatrics, gynaecology and anaesthesia) in order to be able to practice routines and task assignments as realistically as possible.

ii. The number of participants within a scenario shall be adapted to the content (e.g. respiratory problem, resuscitation) and setting (e.g. intensive care unit, trauma room, operating room) of the scenarios. The aim is that the human resources within a training situation are deployed in keeping with reality.[35,36]

iii. In the scenario each participant shall adopt the role of his actual personal and professional position and level of experience. In exceptional cases, it may be useful to change roles in order to gain a better understanding of a different position. However, this is in fact a specific learning objective of the scenario in that case.

iv. The group of participants should be complete from start to finish of the training, and in any case from start to finish of a scenario including debriefing, to ensure psychological safety and integrity.

v. Each participant shall at least once actively participate in a scenario as part of the treating team. The group size of the entire course must be selected accordingly.

vi. Even those persons who are not actively participating in a scenario shall have the opportunity to follow the scenario in the same room or, ideally, via live transmission in a separate room. This allows participants who were not involved in the scenario to participate and learn in the debriefing.

vii. Participants shall be familiar with the official theoretical guidelines / algorithms appropriate to the respective training content (e.g. neonatal resuscitation algorithm for neonatal team training). Participants may be provided with preparatory materials before the start of the training (e.g. self-study using literature, script, e-learning, video links, etc.). Alternatively, or additionally, the simulation training may be preceded by a theoretical part as a refresher. Theoretical content on medical and non-medical topics (NTS/CRM) may be covered here.

viii. Participants shall have already been trained in technical skills such as BLS, bag-mask ventilation etc. before the simulation based team trainings. This may usefully be practised in appropriate training formats (see appendix).

b) SIMULATION TRAINERS

Simulation training shall be conducted with a team of at least two trainers. Ideally, the team of two is supplemented by additional people, e.g. technicians, "SimNurse" and/or additional trainers or debriefers. All these persons shall be familiar with the principles of simulation and with the "Healthcare Simulationist Code of Ethics"[37] (www.netzwerk-kindersimulation.org/wp-content/uploads/2019/05/ Ethikkodex_German-201905.pdf).

i. Debriefers

The debriefing should ideally be conducted by two people. It would be desirable to have debriefers from different professional groups and/or medical disciplines in order to be able to incorporate the respective expertise and different perspectives into the analysis. In addition, a second debriefer offers the advantage of being able to provide support in difficult situations.[38] Following the training, there shall be a mutual feedback between the debriefers in order to facilitate a continuous development. For learning purposes and quality control, regular debriefings on the debriefing should be conducted by experienced instructors who are not involved in the training or debriefing.

ii. Role-players in the scenario (extra, actor, confederate)

In each scenario one person should be available to act as a link between trainers and participants ("confederate").[4,39] The person is present in the simulation room during the scenario and does not participate in the care of the patient without being requested to do so. The person may provide anamnestic information or provide assistance if the participants do not recognise relevant facts or changes in the situation (so-called "life saver").[40]

The role-player must be well informed about his predefined role and understand the objective of the scenario in order not to unduly distract the participants. If the simulator is controlled from outside the simulation room, it is advisable that it is connected to the other instructors via a headset in order to be able to receive appropriate instructions.

The participants shall be informed of the role-player's presence before the scenario begins.

iii. "SimNurse"

The person acting as "SimNurse" may also assume the tasks of the role-player. Furthermore, this person supports the team in the preparation and wrap-up activities for the simulation room and scenario. During the scenario the person assists the participants with questions about technology or equipment and pays attention to the safety of the participants as well as to the proper operation of the simulator.

iv. Technician

An additional person should be available to attend to the technical equipment (simulator, microphones, AV system, etc.) during the simulation, as appropriate to the local technical complexity, and who is proficient in all aspects of the installed equipment. This includes the set-up, operation, problem solving and dismantling of the simulation infrastructure.

v. Administration

Additional personnel (e.g. secretarial staff, student assistant) may be assigned to administrative tasks related to the simulation training (registration, invitation letters, evaluation and issuing of certificates). These people do not require any specific simulation training. However, these persons shall be familiar with the basic features of simulation.

7) SCENARIOS

Predefined scenarios are an elementary part of simulation-based team training and essential to achieve learning objectives.

a) LEARNING OBJECTIVES

i. Learning objectives must be clearly defined and correspond in scope and complexity to the target group.

ii. The scope of the learning objectives must be commensurate with the available training time.

iii. In addition to medical knowledge and technical skills, learning objectives from the NTS (interprofessional communication, role allocation, situational awareness, decision making, teamwork) and corresponding triggers shall be incorporated into the scenario development.

iv. The use of helpful cues or stressors and distractions shall serve to achieve the learning objectives and be adapted to the training level of the target group.

b) SCENARIO DEVELOPMENT

i. The various occupational groups that are also involved in the scenario shall be included in the development of scenarios.

ii. In order to achieve the predefined learning objectives it is necessary to design the scenario with contentual, psychological, staffing and material realism.[20,41] Furthermore, the available technical capabilities shall be taken into account for the development of the scenarios.

iii. Scenarios should be subjected to a practical check, e.g. within the trainer team, before they are used with participants for the first time, in order to identify any unforeseen problems.

iv. The use of stressors or distractions (e.g. agitated parents, defective medical equipment) shall be undertaken with caution, as they can distract from learning objectives.

v. Props like x-rays, ultrasound and blood gas analysis shall be prepared as appropriate to the case. Real cases or images from the Internet can be used for this. The deletion of patient-related data and compliance with copyright regulations are indispensable.

vi. Scenarios should be reviewed at regular intervals within the trainer team for their practical suitability, relevance and currency, and adjusted if necessary.

c) SCENARIO SCRIPT

i. The scenarios shall be set out in writing as scripts to ensure reproducibility, achievement of the learning objectives and continuing development, and to enable all trainers to be prepared.

ii. The topics of a script shall include at least the following elements:

- Target group
- Learning objectives – medical and NTS
- Scenario description for the trainer team: situation, locality, role-players involved in the scenario (e.g. father, emergency physician), process sequence of the scenario, triggers for deterioration or improvement, objectives
- Composition of the participant team
- Participant briefing: Who is in the room? Where does the scenario take place? When does the scenario take place? What do we already know about the patient?
- Description of the planned process sequence for the trainer team: vital parameters, triggers and life savers
- Instructions for role-players
- Checklist for the preparation of the scenario: Simulator, medical equipment, required props, documents, x-rays, ECG, clothing, moulages, etc.[42–46]

8) PROCESS SEQUENCE OF A TRAINING

a) PREBRIEFING OF THE TRAINER TEAM

Before the simulation is started, the trainer team shall jointly discuss the scenarios and learning objectives, including possible life savers, as well as the allocation of tasks. This includes introduction to the course, familiarisation with the simulator and simulation environment, briefing the participants in advance of the scenarios, operating the technical equipment, role allocation during the scenario and debriefing.

b) INTRODUCTION

An introduction of trainers and participants shall take place at the beginning of the course. Subsequently, the framework conditions must be established and the process sequence of the training and organisational arrangements (local facilities, time schedule) must be explained. In addition, the expectations of the participants may be ascertained so that they can be addressed in the training to the extent possible.

c) FAMILIARISATION WITH THE SIMULATOR AND SIMULATION ENVIRONMENT

It is important to familiarise the participants with the capabilities (e.g. respiratory assessment, intubation, establishing vascular access, etc.) and the limitations of the simulator (e.g. skin colour, state of consciousness) and the simulation environment (e.g. emergency trolley, medical equipment, alerting pathways, telephones, role-players, etc.).[16] In this context, the participants must be shown how they can obtain information that the simulator cannot render. The participants shall know which human and material resources are available and which "acute" escalation pathways (e.g. resuscitation telephone number) can be used in the in-situ settings.

Familiarisation shall be conducted in a structured manner (e.g. using the ABCDE approach, the neonatal algorithm or a checklist). The participants shall have the opportunity to actively test the relevant simulator functions (e.g. auscultation, pulses, defibrillation, monitoring, etc.) and, if necessary, to become familiar with the equipment. In addition, safety-relevant aspects (including medical gases, medication, defibrillation energy) shall be addressed, also for the purpose of self-protection. The scope of familiarisation may be adapted to the simulation experience of the participants.

d) BRIEFING OF PARTICIPANTS (PREBRIEFING)

After assigning the active participants, all participants (active and passive) receive a preliminary information (briefing) for the coming scenario. The briefing contains information about the patient, the setting (emergency department, intensive care unit, time of day, etc.) and the role-players integrated in the scenario (parents, emergency physician).

e) IMPLEMENTING THE SCENARIO

The scenario shall proceed as realistically as possible with regard to its timeline and the change of vital parameters. The participants must be provided with all information necessary for the scenario (medical history, patient chart, X-ray, blood gas analysis, etc.). Physiological parameters, which the simulator cannot reproduce, shall be provided to the participants by alternative means (e.g. state of consciousness by parents, recapillarisation time by "SimNurse").

All necessary (invasive) measures on the simulator, (follow-up) alerting of other staff, communication with relatives etc. shall be actually performed by the participants. The trainers must react to the performed measures and control the simulator and vital parameters realistically in terms of timing (e.g. drop in heart rate after volume administration in case of hypovolaemia).

In cases where the actions of the team in reality would result in the "death" of the simulator and the death of the simulator is not an explicit learning objective, life savers, e.g. cues from role-players or from the control room ("Voice of God") shall be used or the scenario shall be paused or terminated ahead of schedule.

f) DEBRIEFING

Following the simulation scenario, the trainer team shall immediately gather the whole group together for the debriefing in order to capture the emerging discussions of the participants. At the debriefing the focus shall be both on the medical contents and on the NTS[5] and CRM principles. Depending on pre-defined learning objectives, the needs of the participants and the process sequence of the scenario, the weighting between medical and non-medical content may vary.

The debriefing shall be conducted in a structured way according to tried and tested models, e.g. "Debriefing with good judgment"[47] or the PEARLS concept[48].[49] The debriefers shall not act as teachers, but as facilitators.

The debriefing shall ideally comprise the following stages: transition stage (retrieval of emotions), description stage, analysis stage and application stage.[50]

Maintaining a positive and psychologically safe learning environment is essential for effective learning. That means that the participants feel a sense of security not to have to be ashamed of being rejected or sanctioned in some other way when they make mistakes, acknowledge them and communicate their true sentiments, and when they do not know something or ask questions.[51] An important basic assumption here is that all participants are intelligent and competent and want to constantly work towards their personal improvement.[52] Furthermore, a variety of implicit and explicit strategies play an important role here before the debriefing (e.g. sitting in a circle, agreement on confidentiality), during the debriefing (e.g. pausing and listening, being vulnerable and curious) and after the debriefing (recognition, goodwill).[51]

Self-reflection of the participants shall be encouraged and possible solutions shall be worked out together in context. In this process, suitable questioning techniques, e.g. 3B-questioning techniques [53] (advocacy and inquiry), circular questions [54], open question technique shall be applied[55].

Both active and passive participants shall be able to make an input. Selected video sequences may be integrated into the debriefing.[32] However, these shall not be used as video evidence, but shall enable the participants to engage in structured reflection. The time available for this very frequently used form of debriefing should be at least twice as long as for the scenario itself.

We would also refer here to other modalities of debriefing (e.g. "Rapid Cycle Deliberate Practice" [56] or "3D Model of Debriefing: Defusing, Discovering, and Deepening"[57]), which are successfully applied especially in North America.

g) IMPLEMENTATION OF OTHER SCENARIOS AND DEBRIEFINGS

Additional scenarios and debriefings are conducted according to the scope of the course or training.

h) COURSE EVALUATION

By the end of the course, a written evaluation and direct feedback should be provided by the participants.

i) DEBRIEFING OF TRAINER TEAM

Following the simulation (scenarios, debriefing), the trainer team shall have a follow-up discussion and give each other constructive and structured feedback regarding the debriefing. Evaluation instruments such as "Objective Structured Assessment of Debriefing" (OSAD)[58,59] or "Debriefing Assessment for Simulation in Healthcare" (DASH)[60] may be useful for this purpose. A mutual debriefing on the debriefings should be performed regularly in order to support the continued development of the trainers [61].

9) FEEDBACK AND EVALUATION

Feedback of the participants to the instructors (in line with the debriefing in the trainer team) after the simulation training is crucial for the enhancement of the learning success and the quality of future trainings and must therefore be a mandatory requirement.

a) FEEDBACK BY PARTICIPANTS

A course evaluation with possible suggestions for improvement provides the opportunity to review the formal and content-related standard of the simulation trainings and to improve it in future.[62,63]

Feedback from the participants shall be given immediately after the training in order to obtain the most authentic impressions possible. Ideally, it should take the form of a spontaneous direct exchange and may also be conducted in standardised anonymous written form.

Feedback from the participants includes: organisational aspects, professionalism of the trainers, presentation of the teaching content, realism of the scenarios, debriefing, learning environment, relevance of what has been learned to everyday clinical practice and an overall appraisal.

b) DEBRIEFING OF TRAINER TEAM

Following the feedback from the participants, a debriefing of the training shall take place within the trainer team. In addition to the above-mentioned mutual debriefing on the debriefing, the following points shall be evaluated: practicability of the scenarios, technical and organisational problems, direct consequences from participant feedback, satisfaction of the trainers, achievement of the learning objectives as well as possible changes or adjustments to the scenarios and the process sequence of future trainings are discussed.

c) SUMMARY OF THE TRAINING

'In Situ' simulations offer the possibility of a system check of the equipment, structural processes (e.g. checklists, SOP) and of team and communication aspects.

It has proven to be effective to document the contents discussed in the debriefings in written form and to make them available to the staff of the department.

10) APPENDIX

To differentiate simulation-based team training from other training formats, the following table outlines the differences in comparison to other training formats:

	Simulation-based team training	Case-based training and training of advanced measures (ALS)	Basic resuscitation measures (BLS)	Training of technical skills
Trainers	At least 2, optimally 4 persons<br>2 debriefers<br>1 role-player / "SimNurse"<br>1 technician<br>Requirements: (see above)<br>Professional competence<br>Training competence (instructor status)<br>Continuous further training	At least 1 person<br>Requirements:<br>Professional competence:<br>Official emergency course of the ERC (e.g. EPLS) or AHA (e.g. PALS) and in-depth specialist competence in the field of the content to be imparted<br>Training competence:<br>Training for teaching the contents to be imparted<br>Regular trainer activity and continued education in the specialist and didactic domain	At least 1 person<br>Requirements:<br>Training for teaching basic resuscitation measures	At least 1 person<br>Requirements:<br>Training for teaching the technical skills to be learned

Equipment (minimum requirement)	Simulator:<br>ALS or "high fidelity" simulator with vital parameter monitor or standardised patient<br>Medical equipment:<br>Real or at least realistic medical equipment (obligatory: material for airway management, monitoring, vascular access [i.v./i.o.], defibrillator, medication)<br>Other:<br>Optimal: Audio-visual system	Simulator:<br>ALS simulator with vital parameter monitor<br>Medical equipment:<br>Real or realistic medical equipment (at least material for airway management, monitoring, vascular access [i.v./i.o.], defibrillator, possibly medication)	Simulator:<br>Low-fidelity phantom<br>Other:<br>Ideally use of a feedback device [64] (quality of ventilation and/or cardiac massage)	Simulator:<br>Phantom suitable for the skill to be learned, e.g. intubation trainer, lumbar puncture trainer<br>Medical equipment:<br>Real medical equipment
Participants	Interprofessional and possibly interdisciplinary teams with a realistic team constellation<br>If possible, with prior BLS or ALS knowledge	Random composition of participant groups<br>Ideally interprofessional	Random composition of participant groups	Random composition of participant groups
Location / Setting	Simulation centre "off-site" or "in situ"<br>Optimal:<br>1 training room<br>1 debriefing room<br>1 technician room / control room	1 training room<br>Optional:<br>1 debriefing room	1 training room	1 training room

11) LITERATURE

1. Deutsche Gesellschaft für Simulation in der Medizin e. V. (DGSiM e.V.), Mindestanforderungen für Teamtrainings der DGSiM. http://www.dgsim.de/sites/default/files/dgsim_mindestanforderungen_teamtraining_version_1.pdf (2015).

2. Society in Europe for Simulation applied to medicine (SESAM). SESAM Accreditation of Simulation Based Educational Institutions – Principles. https://worldspanmedia.s3.amazonaws.com/media/sesam2/uploads/documents/accreditation/sesam-accreditation-of-simulation-based-educational-institutions-principles-2018.pdf.

3. Sittner, B. J. *et al.* INACSL Standards of Best Practice for simulation: Past, present, and future. *Nurs. Educ. Perspect.* (2015) doi:10.5480/15-1670.

4. Lioce L. (Ed.), Downing D., Chang T.P., Robertson J.M., Anderson M., Diaz D.A., and S. A. E. (Assoc. E. . and the T. and C. W. G. (2020). Healthcare Simulation Dictionary – Second Edition. *Rockville, MD: Agency for Healthcare Research and Quality* AHRQ Publication No. 20-0019 https://www.ahrq.gov/patient-safety/resources/simulation/terms.html.

5. Rhona, F., O'Connor, P. & Crichton, M. D. *Safety at the sharp end: a guide to non-technical skills.* (2008).

6. Rall, M. & Lackner, C. K. Crisis Resource Management (CRM)Crisis resource management (CRM). *Notfall + Rettungsmedizin* (2010) doi:10.1007/s10049-009-1271-5.

7. Kanki, B. G. Communication and Crew Resource Management. in *Crew Resource Management* (2010). doi:10.1016/B978-0-12-374946-8.10004-4.

8. Eppich, W. 'Speaking Up' for Patient Safety in the Pediatric Emergency Department. *Clin. Pediatr. Emerg. Med.* (2015) doi:10.1016/j.cpem.2015.04.010.

9. Schmutz, J. B., Lei, Z., Eppich, W. J. & Manser, T. Reflection in the heat of the moment: The role of in-action team reflexivity in health care emergency teams. *J. Organ. Behav.* (2018) doi:10.1002/job.2299.

10. Schmutz, J. B., Kolbe, M. & Eppich, W. J. Twelve tips for integrating team reflexivity into your simulation-based team training. *Med. Teach.* (2018) doi:10.1080/0142159X.2018.1464135.

11. Salas, E., Sims, D. E. & Shawn Burke, C. Is there A 'big five' in teamwork? *Small Group Research* (2005) doi:10.1177/1046496405277134.

12. El-Shafy, I. A. *et al.* Closed-Loop Communication Improves Task Completion in Pediatric Trauma Resuscitation. *J. Surg. Educ.* (2018) doi:10.1016/j.jsurg.2017.06.025.

13. Topping, A. *et al.* Towards identifying nurse educator competencies required for simulation-based learning: A systemised rapid review and synthesis. *Nurse Education Today* (2015) doi:10.1016/j.nedt.2015.06.003.

14. Roh, Y. S. & Jang, K. I. Survey of factors influencing learner engagement with simulation debriefing among nursing students. *Nurs. Heal. Sci.* (2017) doi:10.1111/nhs.12371.

15. Edmondson, A. Psychological safety and learning behavior in work teams. *Adm. Sci. Q.* (1999) doi:10.2307/2666999.

16. Rudolph, J. W., Raemer, D. B. & Simon, R. Establishing a safe container for learning in simulation the role of the presimulation briefing. *Simulation in Healthcare* (2014) doi:10.1097/SIH.0000000000000047.

17. Calhoun, A. W. & Gaba, D. M. Live or Let Die. *Simulation in Healthcare* (2017) doi:10.1097/SIH.0000000000000256.

18. McBride, M. E., Schinasi, D. A., Moga, M. A., Tripathy, S. & Calhoun, A. Death of a simulated pediatric patient: Toward a more robust theoretical framework. *Simul. Healthc.* (2017) doi:10.1097/SIH.0000000000000265.

19. Tripathy, S. *et al.* When the Mannequin Dies, Creation and Exploration of a Theoretical Framework Using a Mixed Methods: Approach. *Simul. Healthc.* (2016) doi:10.1097/SIH.0000000000000138.

20. Dieckmann, P., Gaba, D. & Rall, M. Deepening the theoretical foundations of patient simulation as social practice. *Simulation in Healthcare* (2007) doi:10.1097/SIH.0b013e3180f637f5.

21. Nanji, K. C., Baca, K. & Raemer, D. B. The effect of an olfactory and visual cue on realism and engagement in a health care simulation experience. *Simul. Healthc.* (2013) doi:10.1097/SIH.0b013e31827d27f9.

22. Appendix 1. https://www.faa.gov/about/initiatives/nsp/ac/media/AC_120-40B-Ch2.pdf.

23. Sørensen, J. L. *et al.* Design of simulation-based medical education and advantages and disadvantages of in situ simulation versus off-site simulation. *BMC Med. Educ.* (2017) doi:10.1186/s12909-016-0838-3.

24. Meurling, L. *et al.* Comparison of high- and low equipment fidelity during paediatric simulation team training: A case control study. *BMC Med. Educ.* (2014) doi:10.1186/1472-6920-14-221.

25. Gu, Y. *et al.* The effect of simulator fidelity on acquiring non-technical skills: a randomized non-inferiority trial. *Can. J. Anesth.* (2017) doi:10.1007/s12630-017-0973-2.

26. Finan, E., Bismilla, Z., Whyte, H. E., LeBlanc, V. & McNamara, P. J. High-fidelity simulator technology may not be superior to traditional low-fidelity equipment for neonatal resuscitation training. *J. Perinatol.* (2012) doi:10.1038/jp.2011.96.

27. Langdorf, M. I. *et al.* High-Fidelity Simulation Enhances ACLS Training. *Teach. Learn. Med.* (2014) doi:10.1080/10401334.2014.910466.

28. Cantrell, M. J. & Deloney, L. A. Integration of Standardized Patients into Simulation. *Anesthesiology Clinics* (2007) doi:10.1016/j.anclin.2007.03.003.

29. Bullough, A. S. *et al.* Obstetric team simulation program challenges. *J. Clin. Anesth.* (2016) doi:10.1016/j.jclinane.2016.08.019.

30. Unver, V. *et al.* Integrating simulation based learning into nursing education programs: Hybrid simulation. *Technol. Heal. Care* (2018) doi:10.3233/THC-170853.

31. Ghi, T. *et al.* The use of a hybrid mannequin for the modern high-fidelity simulation in the labor ward: the Italian experience of the Ecografia Gestione Emergenze Ostetriche

(EGEO) group. *Am. J. Obstet. Gynecol.* (2019) doi:10.1016/j.ajog.2019.07.023.

32. Sawyer, T. *et al.* The effectiveness of video-assisted debriefing versus oral debriefing alone at improving neonatal resuscitation performance a randomized trial. *Simulation in Healthcare* (2012) doi:10.1097/SIH.0b013e3182578eae.

33. Ker, J., Mole, L. & Bradley, P. Early introduction to interprofessional learning: A simulated ward environment. *Med. Educ.* (2003) doi:10.1046/j.1365-2923.2003.01439.x.

34. Vanderbilt, A. A., Pappada, S. M., Stein, H., Harper, D. & Papadimos, T. J. Increasing patient safety with neonates via handoff communication during delivery: a call for interprofessional health care team training across GME and CME. *Adv. Med. Educ. Pract.* (2017) doi:10.2147/amep.s129674.

35. Lim, Y. S., Steinemann, S. & Berg, B. W. Team size impact on assessment of teamwork in simulation-based trauma team training. *Hawaii. J. Med. Public Health* (2014).

36. Kelleher, D. C., Kovler, M. L., Waterhouse, L. J., Carter, E. A. & Burd, R. S. Factors affecting team size and task performance in pediatric trauma resuscitation. *Pediatr. Emerg. Care* (2014) doi:10.1097/PEC.0000000000000106.

37. Ethikkodex. https://www.netzwerk-kindersimulation.org/wp-content/uploads/2019/05/Ethikkodex_German-201905.pdf.

38. Cheng, A. *et al.* Co-debriefing for simulation-based education: A primer for facilitators. *Simulation in Healthcare* (2015) doi:10.1097/SIH.0000000000000077.

39. Sanko, J. S., Shekhter, I., Kyle, R. R., Di Benedetto, S. & Birnbach, D. J. Establishing a convention for acting in healthcare simulation: Merging art and science. *Simulation in Healthcare* (2013) doi:10.1097/SIH.0b013e318293b814.

40. Dieckmann, P., Lippert, A., Glavin, R. & Rall, M. When things do not go as expected: Scenario life savers. *Simul. Healthc.* (2010) doi:10.1097/SIH.0b013e3181e77f74.

41. Rudolph, J. W., Simon, R. & Raemer, D. B. Which reality matters? Questions on the path to high engagement in healthcare simulation. *Simulation in Healthcare* (2007) doi:10.1097/SIH.0b013e31813d1035.

42. Seropian, M. A. General Concepts in Full Scale Simulation: Getting Started. *Anesthesia and Analgesia* (2003) doi:10.1213/01.ANE.0000090152.91261.D9.

43. Alinier, G. Developing high-fidelity health care simulation scenarios: A guide for educators and professionals. *Simul. Gaming* (2011) doi:10.1177/1046878109355683.

44. Benishek, L. E. *et al.* The Template of Events for Applied and Critical Healthcare Simulation (TEACH Sim). *Simul. Healthc. J. Soc. Simul. Healthc.* (2015) doi:10.1097/sih.0000000000000058.

45. Lioce, L. *et al.* Standards of Best Practice: Simulation Standard IX: Simulation Design. *Clin. Simul. Nurs.* (2015) doi:10.1016/j.ecns.2015.03.005.

46. Huffman, J. L., McNeil, G., Bismilla, Z. & Lai, A. Essentials of Scenario Building for Simulation- Based Education. in (2016). doi:10.1007/978-3-319-24187-6_2.

47. Rudolph, J. W., Simon, R., Rivard, P., Dufresne, R. L. & Raemer, D. B. Debriefing with Good Judgment: Combining Rigorous Feedback with Genuine Inquiry. *Anesthesiology Clinics* (2007) doi:10.1016/j.anclin.2007.03.007.

48. Eppich, W. & Cheng, A. Promoting excellence and reflective learning in simulation (PEARLS): Development and rationale for a blended approach to health care simulation debriefing. *Simul. Healthc.* 10, 106–115 (2015).

49. Sawyer, T., Eppich, W., Brett-Fleegler, M., Grant, V. & Cheng, A. More Than One Way to Debrief. *Simul. Healthc. J. Soc. Simul. Healthc.* (2016) doi:10.1097/sih.0000000000000148.

50. Eppich, W. & Cheng, A. Promoting Excellence and Reflective Learning in Simulation (PEARLS). *Simul. Healthc. J. Soc. Simul. Healthc.* (2015) doi:10.1097/sih.0000000000000072.

51. Kolbe, M. *et al.* Managing psychological safety in debriefings: A dynamic balancing act. *BMJ Simul. Technol. Enhanc. Learn.* (2019) doi:10.1136/bmjstel-2019-000470.

52. Rudolph, J. W., Simon, R., Raemer, D. B. & Eppich, W. J. Debriefing as formative assessment: Closing performance gaps in medical education. in *Academic Emergency Medicine* (2008). doi:10.1111/j.1553-2712.2008.00248.x.

53. Rudolph, J. W., Simon, R., Dufresne, R. L. & Raemer, D. B. There's no such thing as 'nonjudgmental' debriefing: a theory and method for debriefing with good judgment. *Simul. Healthc.* (2006) doi:10.1097/01266021-200600110-00006.

54. Kolbe, M., Marty, A., Seelandt, J. & Grande, B. How to debrief teamwork interactions: using circular questions to explore and change team interaction patterns. *Adv. Simul.* (2016) doi:10.1186/s41077-016-0029-7.

55. Dieckmann, P., Friis, S. M., Lippert, A. & Østergaard, D. The art and science of debriefing in simulation: Ideal and practice. *Med. Teach.* (2009) doi:10.1080/01421590902866218.

56. Hunt, E. A. *et al.* Pediatric resident resuscitation skills improve after 'Rapid Cycle Deliberate Practice' training. *Resuscitation* (2014) doi:10.1016/j.resuscitation.2014.02.025.

57. Zigmont, J. J., Kappus, L. J. & Sudikoff, S. N. The 3D Model of Debriefing: Defusing, Discovering, and Deepening. *Seminars in Perinatology* (2011) doi:10.1053/j.semperi.2011.01.003.

58. Runnacles, J., Thomas, L., Sevdalis, N., Kneebone, R. & Arora, S. Development of a tool to improve performance debriefing and learning: The paediatric Objective Structured Assessment of Debriefing (OSAD) tool. *Postgrad. Med. J.* (2014) doi:10.1136/postgradmedj-2012-131676.

59. Runnacles, J., Thomas, L., Korndorffer, J., Arora, S. & Sevdalis, N. Validation evidence of the paediatric Objective Structured Assessment of Debriefing (OSAD) tool. *BMJ Simul. Technol. Enhanc. Learn.* (2016) doi:10.1136/bmjstel-2015-000017.

60. DASH. https://harvardmedsim.org/debriefing-assessment-for-simulation-in-healthcare-dash-german/.

61. Cheng, A. *et al.* Coaching the Debriefer. *Simul. Healthc.* (2017) doi:10.1097/SIH.0000000000000232.

62. McOwen, K. S., Bellini, L. M., Morrison, G. & Shea, J. A. The development and implementation of a health-system-wide evaluation system for education activities: Build it and they will come. *Academic Medicine* (2009)

doi:10.1097/ACM.0b013e3181b6c996.

63. Schiekirka, S. *et al.* Student perceptions of evaluation in undergraduate medical education: A qualitative study from one medical school. *BMC Med. Educ.* (2012) doi:10.1186/1472-6920-12-45.

64. Wagner, M. *et al.* Effects of feedback on chest compression quality: A randomized simulation study. *Pediatrics* 143, (2019).
